# Scleractinian corals (Fungiidae, Agariciidae and Euphylliidae) of Pulau Layang-Layang, Spratly Islands, with a note on *Pavona maldivensis* (Gardiner, 1905)

**DOI:** 10.3897/zookeys.517.9308

**Published:** 2015-08-12

**Authors:** Zarinah Waheed, Francesca Benzoni, Sancia E.T. van der Meij, Tullia I. Terraneo, Bert W. Hoeksema

**Affiliations:** 1 Department of Marine Zoology, Naturalis Biodiversity Center, P.O. Box 9517, 2300 RA Leiden, The Netherlands; 2 Borneo Marine Research Institute, Universiti Malaysia Sabah, Jalan UMS, 88400 Kota Kinabalu, Sabah, Malaysia; 3 Department of Biotechnology and Biosciences, University of Milano-Bicocca, Piazza della Scienza 2, 20126 Milan, Italy; 4 Red Sea Research Center, King Abdullah University of Science and Technology (KAUST), Thuwal, Saudi Arabia

**Keywords:** Scleractinia, South China Sea, Malaysia, atoll, distribution ranges, new records, species richness

## Abstract

Layang-Layang is a small island part of an oceanic atoll in the Spratly Islands off Sabah, Malaysia. As the reef coral fauna in this part of the South China Sea is poorly known, a survey was carried out in 2013 to study the species composition of the scleractinian coral families Fungiidae, Agariciidae and Euphylliidae. A total of 56 species was recorded. The addition of three previously reported coral species brings the total to 59, consisting of 32 Fungiidae, 22 Agariciidae, and five Euphylliidae. Of these, 32 species are new records for Layang-Layang, which include five rarely reported species, i.e., the fungiids *Lithophyllon ranjithi*, *Podabacia sinai*, *Sandalolitha boucheti*, and the agariciids *Leptoseris kalayaanensis* and *Leptoseris troglodyta*. The coral fauna of Layang-Layang is poor compared to other areas in Sabah, which may be related to its recovery from a crown-of-thorns seastar outbreak in 2010, and its low habitat diversity, which is dominated by reef slopes consisting of steep outer walls. Based on integrative molecular and morphological analyses, a *Pavona* variety with small and extremely thin coralla was revealed as *Pavona maldivensis*. Since specimens from Sabah previously identified as *Pavona maldivensis* were found to belong to *Pavona explanulata*, the affinities and distinctions of *Pavona maldivensis* and *Pavona explanulata* are discussed.

## Introduction

Pulau [island] Layang-Layang is a small island standing 2 m high on the southeast rim of a reef known as Swallow Reef (Hancox and Prescott 1995). The reef is an atoll situated at the southern edge of the Spratly Islands in the South China Sea, approximately 300 km northwest of Kota Kinabalu, Sabah, Malaysia. Layang-Layang was reported to have one of the best reefs in East Malaysia in terms of coral cover and diversity and fish life (Ismail et al. 1998; Pilcher and Cabanban 2000).

Being remote, the reef was regarded to be in pristine condition (Pilcher et al. 1999; Pilcher and Cabanban 2000; Zainuddin et al. 2000), although it had experienced disturbances in the past years. In the 1980s, reclamation work was carried out to accommodate a military base, and in the 1990s the island was further extended to construct an airstrip, a resort and a seawall. For the latter developments, coral and sand were mined from the lagoon for building material. A comparative study in 1993 and 1998 showed that the impact of the development on the reef was most evident in the lagoon, with a reduction on live coral cover from 29% to 10% (Mohamed et al. 1994; Zakariah et al. 2007). The outer reefs were not affected by the construction development, except at sites immediate to the island. Here the coral cover averaged 48% at 5 m and 34% at 10 m depth in 1993 (Mohamed et al. 1994). At subsequent independent surveys from 1996 to 1999 in four outer reef sites, mean live coral cover was 73% at 5 m and 58% at 10 m depth (Pilcher and Cabanban 2000).

A massive coral bleaching event during the 1997–98 El Niño event had also affected the reefs of Layang-Layang. Up to 40% of the coral colonies at less than 10 m depth and 25% at 10-20 m depth were bleached at 55 monitoring sites, but by 1999 the corals had recovered or were overgrown with zoantharians and soft corals (Pilcher and Cabanban 2000).

A recent calamity to confront the reefs was an outbreak of the crown-of-thorns (COT) seastar, *Acanthaster planci* (Linnaeus, 1758), in July 2010. During a 3-day survey, densities of 1,011 COTs were counted in a 7,000 m^2^ reef area over eight sites, which corresponds to over 1,400 individuals per ha with dominant size class of 21-30 cm (Nasrulhakim et al. 2010). During the time of the survey, reefs in the southwest of the atoll were badly damaged and had dead corals covered by algae. Although damage to the reefs was not quantified, it was noted that COT had started to infest the reefs in the northwest of the atoll and coral mortality was not as extensive as compared to the reefs in the southwest (Nasrulhakim et al. 2010).

While several short research expeditions and surveys have been carried out to collect baseline information on the marine biodiversity of Pulau Layang-Layang (Zakariah et al. 2007), only one checklist of hard coral species is available as reference, with over 140 species reported by Ridzuan et al. (n.d.) cited in Pilcher and Cabanban (2000) (pp 46–47, Suppl. material VI). In the present study, we aim to update the species list of the hard coral families Fungiidae, Agariciidae and Euphylliidae in Layang-Layang, as similarly done for the reefs of Sabah, Malaysia (Waheed and Hoeksema 2013, 2014). These families, together consisting of ~100 species, were selected as a proxy for scleractinian reef coral diversity, as they can be found in a variety of reef habitats and in a wide geographical range within the Indo-Pacific (Veron 2000). At the time of the survey, small, thin and encrusting corals thought to belong to an unknown *Pavona* species were encountered. Several specimens that were collected for closer inspection of corallite morphology appeared to match with *Pavona maldivensis* (Gardiner, 1905) despite the unusual growth form of the corallum. For verification, a comparison was made between these *Pavona maldivensis* specimens and those collected from other localities including its type locality in the Maldives. Samples of *Pavona explanulata* (Lamarck, 1816) closely resembling *Pavona maldivensis* were also examined in order to better define the boundaries between these two species.

## Methods

### Physical setting

Pulau Layang-Layang (7°22'20"N, 113°50'30"E) measures approximately 1,500 m × 200 m (Google Earth 2013). The only infrastructures on the island are buildings of the Royal Malaysian Navy base, the Marine Research Station Layang-Layang (MARSAL) of the Fisheries Department and the Avillion Layang-Layang Resort. An airstrip runs alongside these establishments. The atoll is somewhat oval in shape situated in a SW-NE axis and measures approximately 7 km long and 2 km wide. Its rim is formed by a ring of 13 shallow reefs, which covers an area of over 4 km^2^ (Musa et al. 2006). The reef circumference is almost 17 km with a sandy cove at the western end and it encloses a shallow lagoon with a maximum depth of 20 m (Pilcher et al. 1999, Sahari et al. 2004, Svrcula 2008). The reefs rise to sea level from around 1,500 m depth forming steep outer reef walls (see Hutchison and Vijayan 2010). The north and northeast reef slopes have a more gradual profile to depths of 20–25 m before plunging down steeply, as compared to the reefs in the south and southwest where the reefs form vertical walls.

Water parameters were measured at 10 m depth of each survey site (Suppl. material 1). The water temperature ranged 28.4–30.0 °C, with a salinity range of 30.1–31.2 ppt. Temperature and salinity measurements were slightly higher in July 2002 (see Ku Yaacob and Ibrahim 2004) in comparison to our readings in March 2013.

Layang-Layang in the South China Sea is influenced by the monsoon system (see Wyrtki 1961). The northeast monsoon dominates between November to March and the southwest monsoon prevails from May to September (Saadon et al. 1999, Morton and Blackmore 2001, Ku Yaacob and Ibrahim 2004, Akhir 2012) while the transitional periods are in April and October (Saadon et al. 1999). The surface current patterns are characterised by the monsoonal system (Akhir 2012). The northeast monsoon causes an anticlockwise circulation pattern in the South China Sea, creating a southwesterly current from the northern rim of the sea, which either departs via the Karimata Straits or turns northeasterly along the west coast of Borneo (East Malaysia) and Palawan, whereas the southwest monsoon reverses the current direction, driving a northward current in the central South China Sea while creating a clockwise gyre above the Spratly Islands (Wyrtki 1961, Morton and Blackmore 2001). The rainy season occurs during the northeast monsoon and due to unpredictable weather Layang-Layang is not accessible for diving. The diving season lasts from March to September annually.

### Field sampling

**Figure 1.**
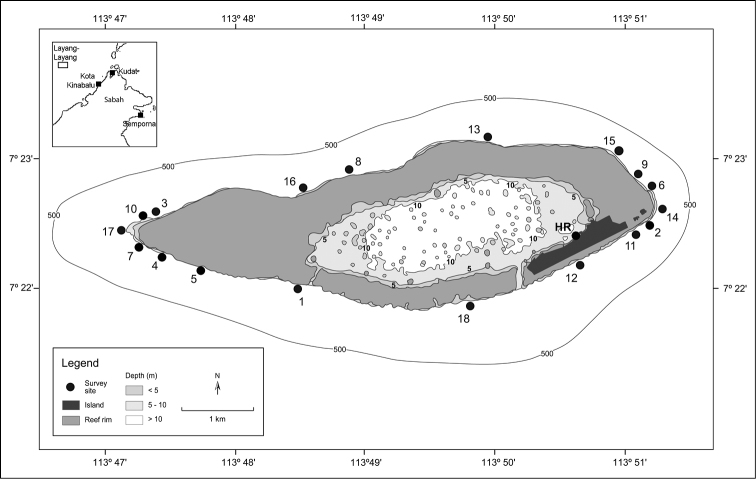
Research area at Pulau Layang-Layang, Spratly Islands. Survey sites are numbered 1-18, and HR for the House Reef. Depth contours are indicated in metres (smaller font). For a complete georeferenced list of localities and depths of survey sites, see Table 1.

Fieldwork was carried out 24–30 March 2013 on the reefs of Layang-Layang. Due to safety issues, surveys were confined to dive sites designated for tourism around the atoll. A total of 18 sites was surveyed on the outer slope of the reef wall from a maximum depth of 40 m to the shallow reef crest of 1 m using the roving diver technique (Schmitt et al. 2002) (Figure 1, Table 1). An additional dive was made at the House Reef (10 m maximum depth) off the resort jetty (7°22'23"N, 113°50'37"E). A checklist of the coral families Fungiidae (sensu Gittenberger et al. 2011, Benzoni et al. 2012a), Agariciidae and Euphylliidae (sensu Veron 2000) was made for each site with photo documentation of each species. Specimens that could not be identified *in situ* were collected for further examination and are kept at the Borneo Marine Research Institute reference collection, Universiti Malaysia Sabah (UMS) in Kota Kinabalu.

**Table 1.** Locality data of survey sites at Pulau Layang-Layang, Spratly Islands.

Site	Site name	Latitude (N)	Longitude (E)	Date	Max. depth (m)
1	Snapper Ledge	07°22.048	113°48.467	24/03/2013	31.8
2	Dogtooth Lair	07°22.470	113°51.100	24/03/2013	30.7
3	Wrasse Strip	07°22.557	113°47.397	25/03/2013	30.3
4	Shark Cave 1	07°22.279	113°47.457	25/03/2013	32.6
5	D’Wall	07°22.191	113°47.701	25/03/2013	33.8
6	Gorgonian Forest	07°22.710	113°51.218	26/03/2013	37.9
7	Shark Cave 2	07°22.323	113°47.321	26/03/2013	27.9
8	Crack Reef	07°22.876	113°48.910	26/03/2013	29.3
9	Coral Café	07°22.773	113°51.144	27/03/2013	38.5
10	Wrasse Strip 2	07°22.555	113°47.371	27/03/2013	33.5
11	Wreck Point	07°22.407	113°51.032	27/03/2013	33.3
12	Wreck Point 2	07°22.197	113°50.649	28/03/2013	40.4
13	Navigator Lane	07°23.110	113°49.979	28/03/2013	36.8
14	The Point	07°22.573	113°51.254	28/03/2013	34.2
15	Coral Café 2	07°23.013	113°50.912	29/03/2013	36.7
16	Mid Reef	07°22.725	113°48.539	29/03/2013	37.7
17	The Valley	07°22.447	113°47.180	29/03/2013	34.8
18	Runway	07°21.902	113°49.778	30/03/2013	40.3

Coral specimens were identified by referring to taxonomic literature (Dinesen 1980, Veron and Pichon 1980, Hoeksema 1989, 2012a, 2012b, 2014, Veron 2000, Ditlev 2003, Licuanan and Aliño 2009, Gittenberger et al. 2011, Benzoni et al. 2012a). Recent molecular studies have led to taxonomic revisions of many scleractinian corals, including the families Agariciidae and Euphylliidae (sensu Veron 2000). The genera *Coeloseris*, *Pachyseris*, *Catalaphyllia*, *Nemenzophyllia*, *Physogyra* and *Plerogyra* are now classified *incertae sedis* (Fukami et al. 2008, Kitahara et al. 2010, Benzoni et al. 2014). Nevertheless, these genera were included in the checklist for comparison with similar studies previously conducted around Sabah (Waheed and Hoeksema 2013, 2014, Waheed et al. subm).

Specimens of a thin morph of *Pavona maldivensis* were collected and small fragments were preserved in 95% absolute ethanol for molecular analyses. The specimens were bleached with sodium hypochlorite, rinsed, air-dried and small fragments were taken for morphological examination. The remaining coralla of these specimens are kept in the dry reference collection of the Borneo Marine Research Institute, UMS.

### Further examination of *Pavona* corals

Seven samples of *Pavona maldivensis* corals collected from Layang-Layang and samples from Banggi, North Sabah (n=1), Ternate, Indonesia (n=1), New Caledonia (n=2) and the Maldives (n=2) were used for further molecular and morphological analyses. Samples of *Pavona explanulata* collected from Banggi, North Sabah (n=1), Ternate, Indonesia (n=2) and Redang, Peninsular Malaysia (n=1) closely resembling *Pavona maldivensis* were also included. In total, 17 samples were used in the analyses (Suppl. material 2).

### Molecular analyses

Coral samples were sequenced for two markers, namely the mitochondrial intergenic spacer between CO1 and 16S-rRna (IGR for short; Terraneo et al. 2014) and the nuclear internal transcribed spacers 1 and 2 including the 5.8S region (ITS for short; White et al. 1990, Takabayashi et al. 1998). DNA extraction was performed using the DNeasy Blood and Tissue Kit (QIAGEN) following the manufacturer’s protocol for animal tissue. The samples were left to incubate overnight. The extracts had concentrations of between 1 to 3 ng/µl for the PCR, quantified using a NanoDrop ND-1000 Spectrophotometer. The PCR mixture was composed of 2.5 µl CoralLoad Buffer (containing 15 mM MgCl_2_), 1.0 µl of each primer (10 pmol), 0.5 µl dNTPs (2.5 mM), 0.5 µl Taq polymerase (15 units/ µl), 18.5 µl of extra pure water and 1.0 µl DNA extract. The primer sequences and PCR amplification details are provided in Table 2. The PCR cycles consisted of an initial denaturation step of 95 °C for 2 min, followed by 39 cycles of 95 °C for 30 s, annealing temperature for 1 min, extension step of 72 °C for 1 min and a final elongation step of 72 °C for 5 min. The PCR products were run on a 1% agarose gel electrophoresis, stained with ethidium bromide and visualized on a Red^TM^ Personal Imaging System. Successfully amplified samples were sent to Macrogen Europe for bidirectional sequencing on an ABI Automated Sequencher 3730xl. The sequences were edited and assembled with Sequencher 4.10.1 and the consensus sequences were blasted against GenBank to check for specific amplification or contamination.

**Table 2.** Primer pairs, gene region, fragment size, annealing temperature and references of the molecular markers used in this study.

Name	Primer	Gene region	Fragment size	Annealing temp.	Reference
AGAH	GCT TGA CAG GGT TTC CAA GA	COI-1-rRNA intron	~1200	54 °C	Terraneo et al. (2014)
AGAL	CGC ATT GAA ACA CGA GCT TA	COI-1-rRNA intron	~1200	54 °C	Terraneo et al. (2014)
ITS4	CCT CCG CTT ATT GAT ATG C	ITS1-5.8S-ITS2	~700	55 °C	White et al. (1990)
A18S	GAT CGA ACG GTT TAG TGA GG	ITS1-5.8S-ITS2	~700	55 °C	Takabayashi et al. (1998)

Sequences were aligned on the GUIDANCE server using PRANK algorithm (Penn et al. 2010a, b) and pruned in BioEdit 7.2.5 (Hall 1999). Gaps were treated as missing data. Pairwise genetic differences were calculated as uncorrected p-distance in MEGA 6.06 (Tamura et al. 2013). The most appropriate model of nucleotide substitution based on the Akaike Information Criterion (AIC) as determined in jModelTest 2.1.6 (Darriba et al. 2012) was a three-parameter model a proportion of invariant sites (TPM3uf+I) for IGR and a Kimura two-parameter model with a proportion of invariant sites and gamma distributed rates (K80+I+G) for ITS. Phylogenies were reconstructed separately for each marker and for the concatenated dataset partitioned by genes based on three optimality criteria.

Maximum Likelihood (ML) analyses were carried out in Garli 2.0 (Zwickl 2006) with the default configuration settings. Separate runs were made for searching the ML tree (100 replicates of random addition) and bootstrapping (1000 replicates). The bootstrap consensus tree was visualised with SumTrees 3.3.1 of the DendroPy 3.12.0 package (Sukumaran and Holder 2010) with a majority rule consensus that includes branch length information. Maximum Parsimony (MP) analyses were conducted in PAUP* 4.0a136 (Swofford 2002) using heuristic searches with 100 replicates of random addition with a Tree Bisection and Reconnection (TBR) branch swapping method. Branch support was obtained with 1000 bootstrap replicates to produce a majority rule consensus tree. Bayesian Inferences (BI) were made in MrBayes 3.2.2 (Huelsenbeck and Ronquist 2001, Ronquist and Huelsenbeck 2003, Ronquist et al. 2012), whereby four Markov Chain Monte Carlo (MCMC) of 10 million generations were applied in two runs, saving one tree every 100 generations and discarding the initial 25% of the total trees as burnin. The average standard deviation of split frequencies after 10 million generations was 0.001615 for IGR, 0.001679 for ITS and 0.001840 for the concatenated dataset in the Bayesian analyses. For the mtDNA phylogeny, sequences of *Pavona maldivensis* and *Pavona explanulata* available on GenBank (Luck et al. 2013) were included in the analyses. In order to root the trees, the closely related species *Leptoseris foliosa* was selected as outgroup (Benzoni et al. 2012b, Terraneo et al. 2014). Novel sequences were submitted to GenBank (accession numbers KR706116–KR706143).

### Morphological analyses

A subset of the *Pavona* corals was examined under a Leica MZ16 microscope and analysed using scanning electron microscope (SEM). Coral fragments were mounted on SEM stubs using blu-tack and coated with Pd/Au for 8 minutes. Images were taken with a JEOL JSM6490LV scanning electron microscope. Distinguishing characters for species identification include macromorphological features of the corallum and calices as well as micromorphological features of the septocostae, columella and radial elements (terminology according to Dinesen 1980, Budd et al. 2012, Benzoni et al. 2012b). Original species descriptions of *Pavona maldivensis* and *Pavona explanulata* as well as descriptions by Wells (1954), Pillai and Scheer (1976), Scheer and Pillai (1983), Veron and Pichon (1980) were used as references.

## Results

### Coral checklist

**Figure 2.**
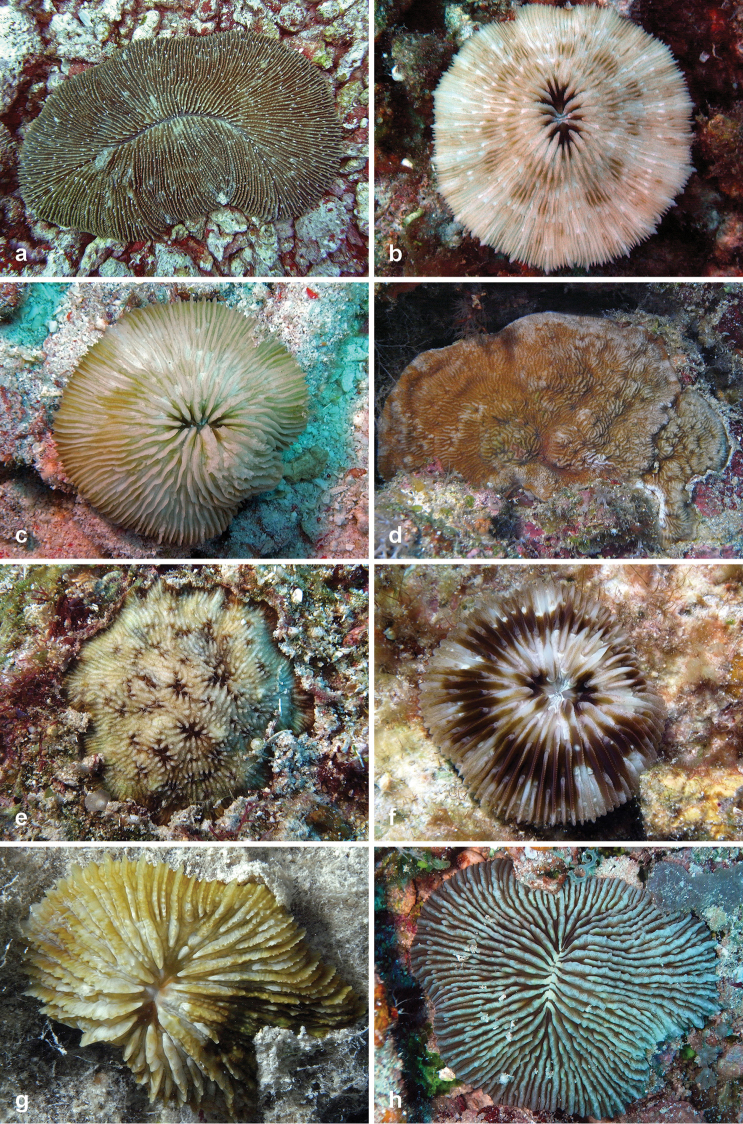
Fungiidae recorded at Pulau Layang-Layang in this study. **a** *Ctenactis albitentaculata* **b** *Cycloseris boschmai* **c** *Cycloseris costulata* **d** *Cycloseris explanulata* **e** *Cycloseris mokai* **f** *Cycloseris tenuis* **g** *Danafungia horrida* **h** *Danafungia scruposa*.

**Figure 3.**
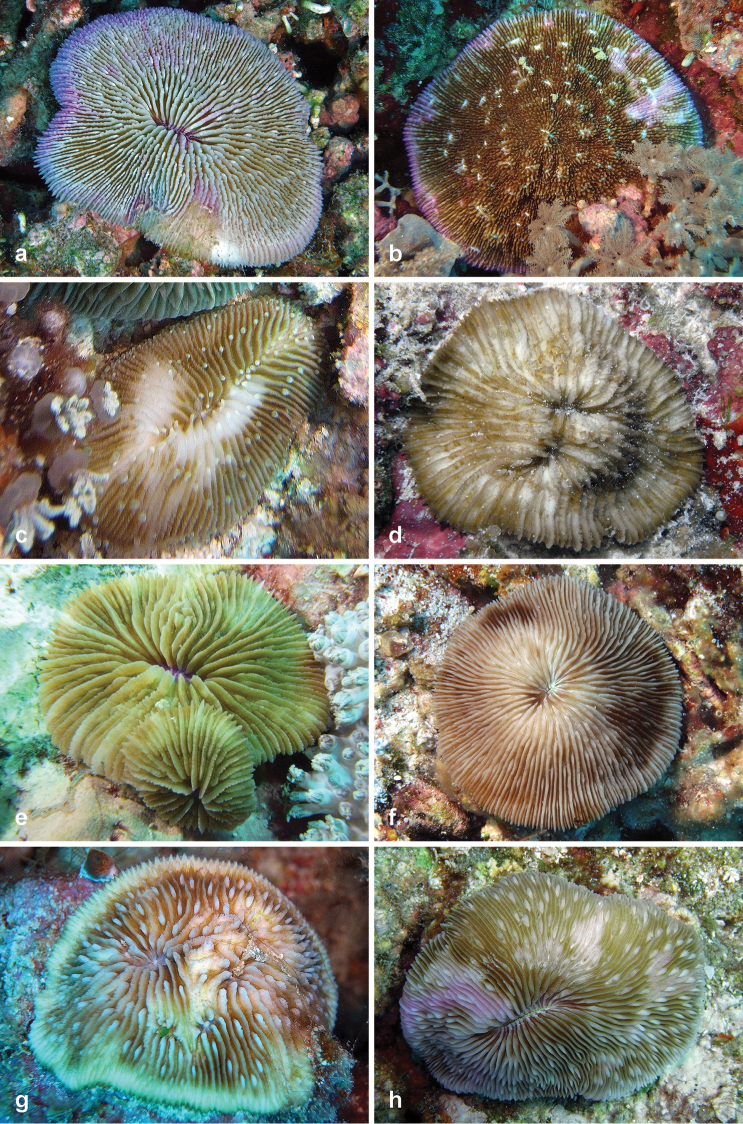
Fungiidae recorded at Pulau Layang-Layang in this study. **a** *Fungia fungites* b *Halomitra pileus* **c** *Herpolitha limax* **d** *Lithophyllon ranjithi* **e** *Lithophyllon repanda* **f** *Lithophyllon scabra* **g** *Lithophyllon undulatum* **h** *Lobactis scutaria*.

**Figure 4.**
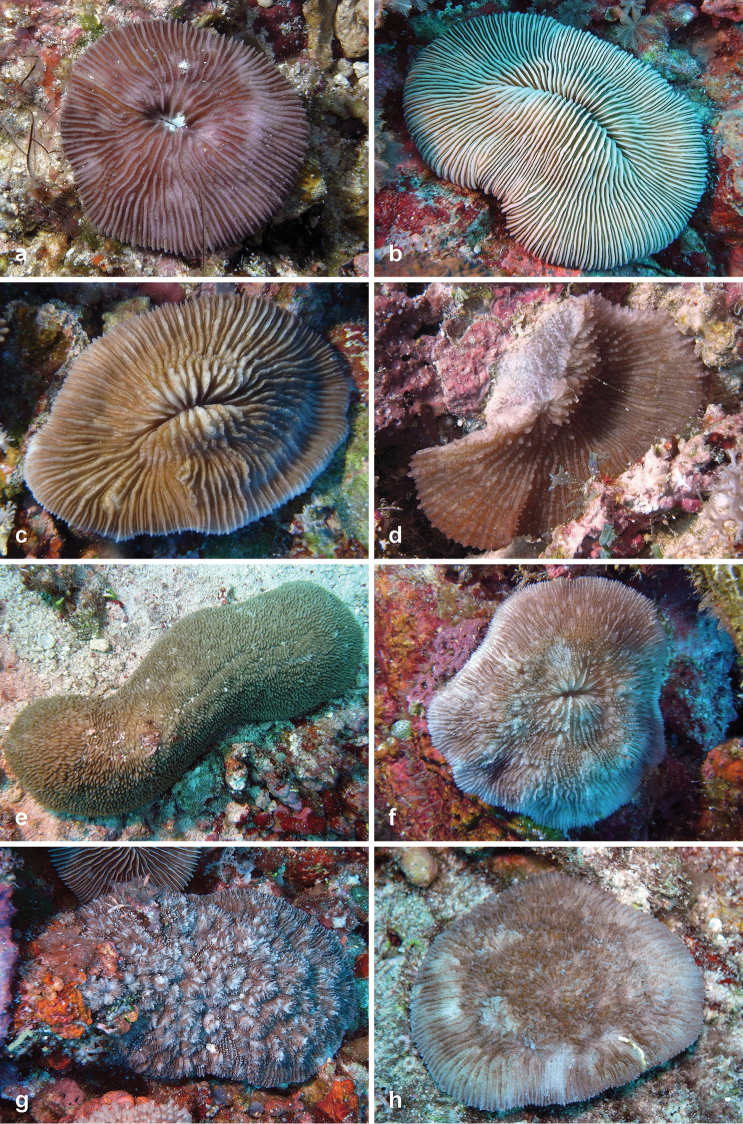
Fungiidae recorded at Pulau Layang-Layang in this study. **a** *Pleuractis granulosa* **b** *Pleuractis gravis* **c** *Pleuractis moluccensis* **d** *Podabacia sinai* **e** *Polyphyllia talpina* **f** *Sandalolitha boucheti* **g** *Sandalolitha dentata* **h** *Sandalolitha robusta*.

**Figure 5.**
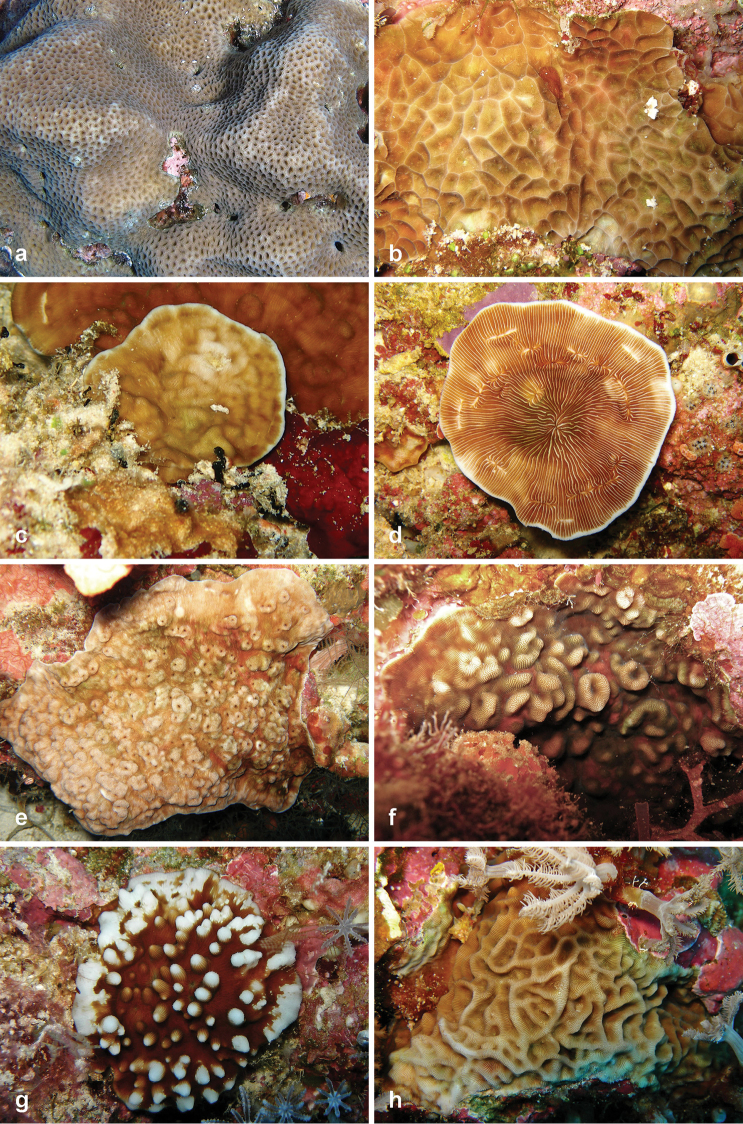
Agariciidae recorded at Pulau Layang-Layang in this study. **a** *Coeloseris mayeri* **b** *Gardineroseris planulata* **c** *Leptoseris foliosa* **d** *Leptoseris glabra* **e** *Leptoseris hawaiiensis* **f** *Leptoseris incrustans* **g** *Leptoseris kalayaanensis* **h** *Leptoseris mycetoseroides*.

**Figure 6.**
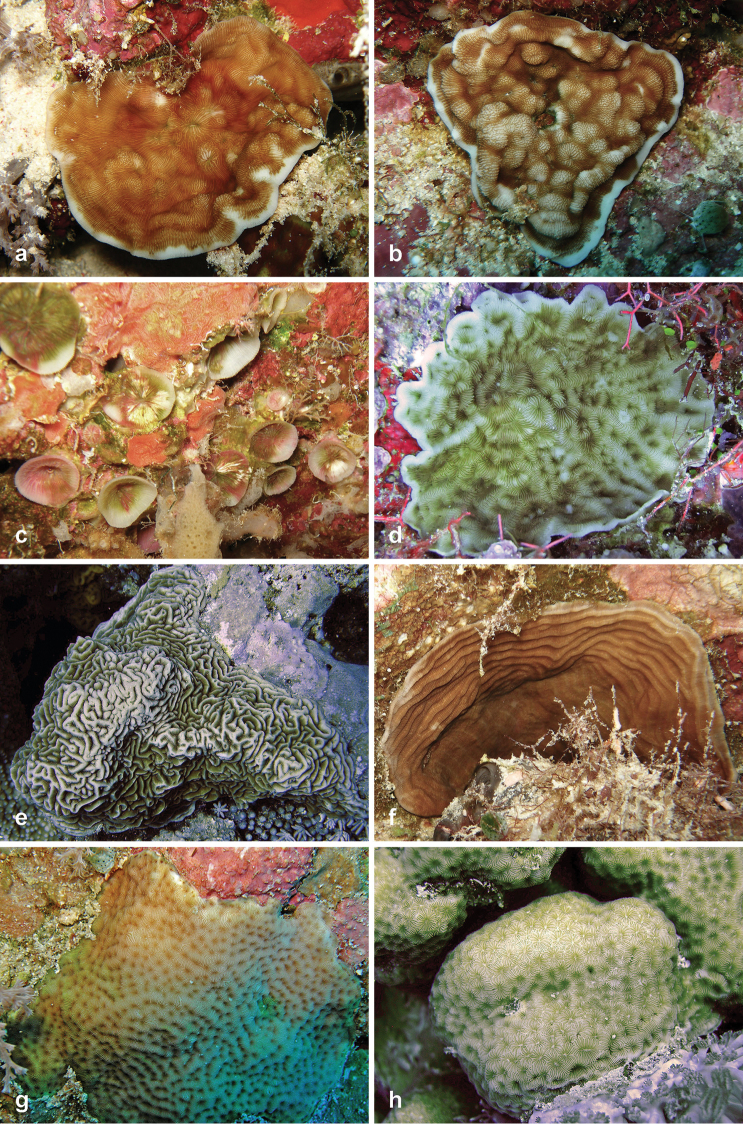
Agariciidae recorded at Pulau Layang-Layang in this study. **a** *Leptoseris scabra* **b** *Leptoseris solida* **c** *Leptoseris troglodyta* **d** *Leptoseris yabei* **e** *Pachyseris rugosa* **f** *Pacyhseris speciosa* **g** *Pavona bipartita* **h** *Pavona clavus*.

**Figure 7.**
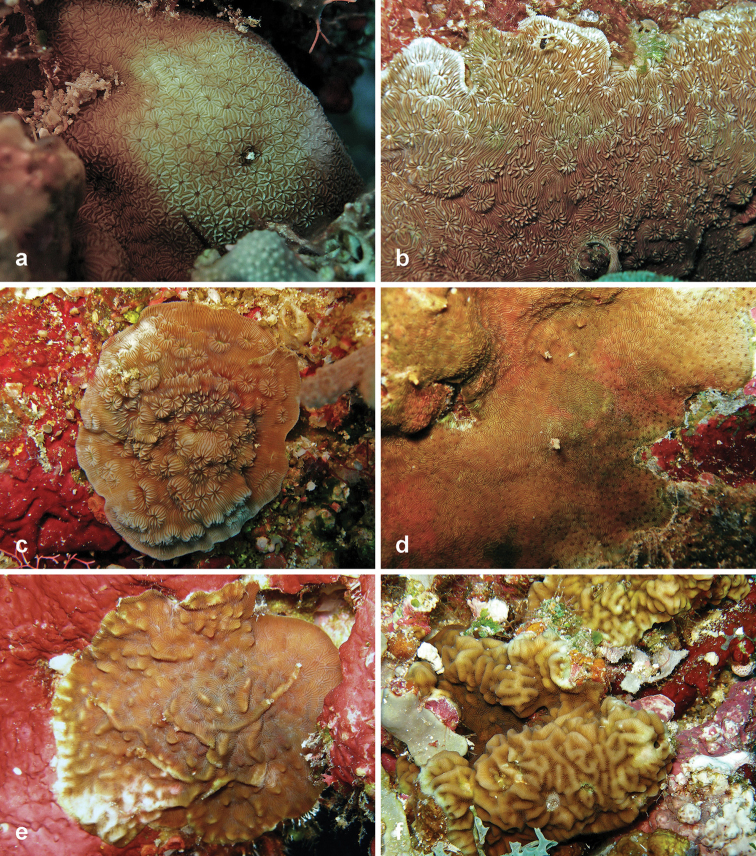
Agariciidae recorded at Pulau Layang-Layang in this study. **a** *Pavona duerdeni* **b** *Pavona explanulata* **c** *Pavona maldivensis* (registration no. IPMB-C 13.00007) **d** *Pavona minuta* **e** *Pavona varians* **f** *Pavona venosa*.

**Figure 8.**
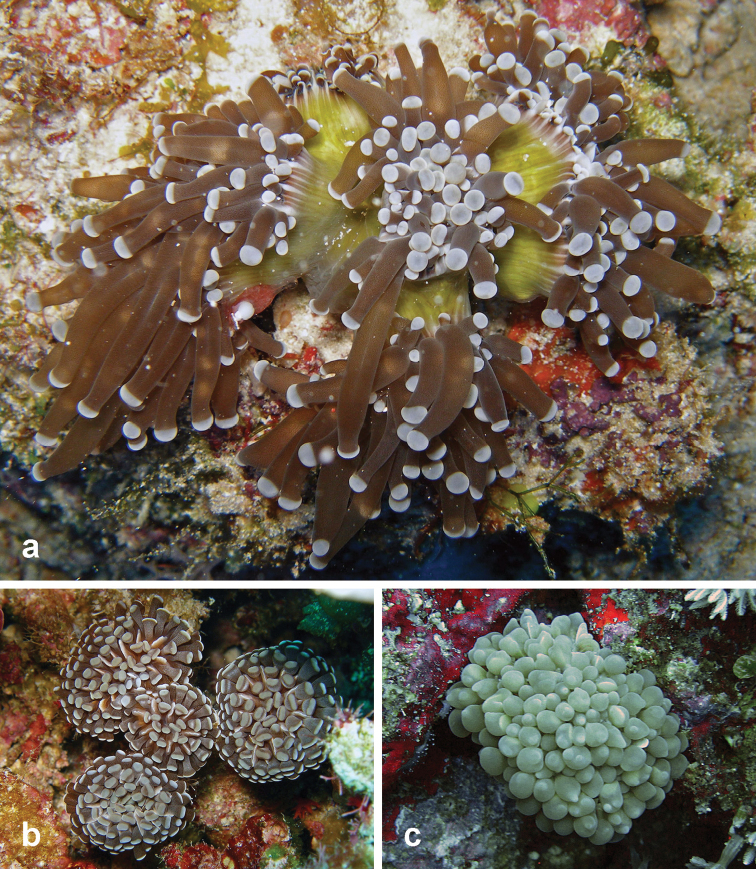
Euphylliidae recorded at Pulau Layang-Layang in this study. **a** *Euphyllia glabrescens* **b** *Euphyllia paraancora* **c** *Physogyra lichtensteini*.

**Figure 9.**
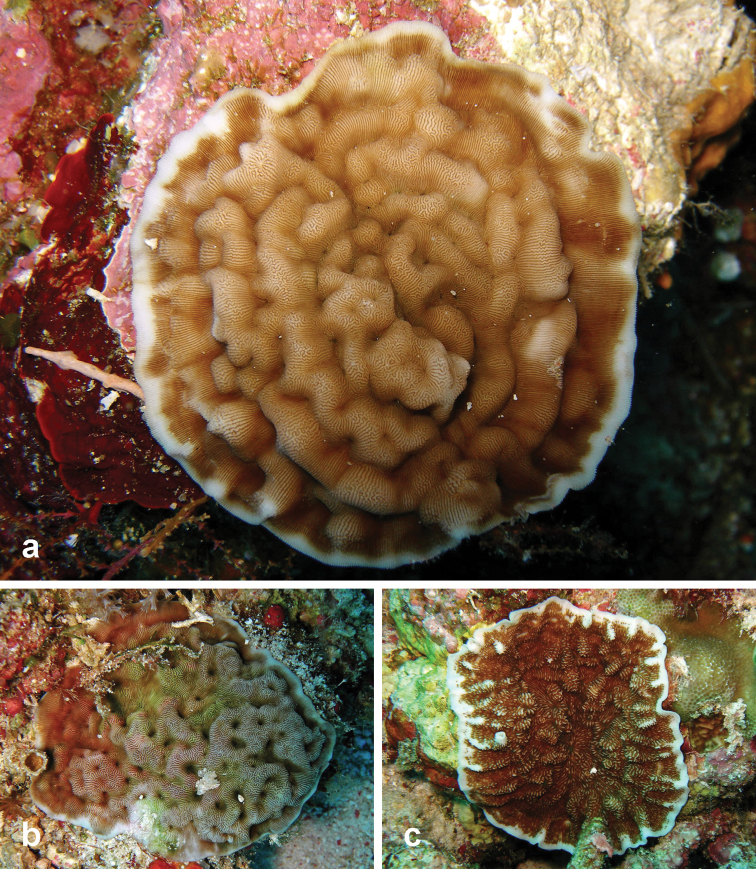
Undetermined *Leptoseris* coral species recorded at Pulau Layang-Layang in this study **a** *Leptoseris* sp. 1 (registration no. IPMB-C 13.00009) **b** *Leptoseris* sp. 2 **c** *Leptoseris* sp. 3.

The number of scleractinian corals recorded in the study area is 56 species with 31 Fungiidae, 22 Agariciidae and three Euphylliidae (Table 3, Figures 2–8, Suppl. material 3). Thirty-two species were not documented from Layang-Layang before and are considered new records (17 Fungiidae, 14 Agariciidae, and one Euphylliidae). Most coral colonies were small in size, making identification difficult for some specimens. Specimens that could not be identified to species level *in situ* include corals of three *Leptoseris* spp. (Figure 9). All were encountered once, except *Leptoseris* sp. 1 at two sites. The House Reef within the lagoon was species-poor in terms of Scleractinia and only one fungiid species, *Danafungia horrida*, was encountered.

**Table 3.** Checklist of hard coral families Fungiidae, Agariciidae and Euphylliidae from 18 sites at Layang-Layang. Species marked with an asterisk (*) are now considered *incertae sedis*. The number of sites indicate the species occurrence frequencies. The figure number corresponds with photos in Figures 2 to 8, while a dash (-) denotes no available photo from the surveys. Previous records are indicated as follows: a = Pilcher and Cabanban (2000), b = Abdullah (2005), dash (-) = species not reported before, therefore are considered new records for Layang-Layang.

Family	No.	Species	No. of sites	Figure no.	Previous records
Fungiidae	1	*Ctenactis albitentaculata* Hoeksema, 1989	1	2a	b
	2	*Ctenactis crassa* (Dana, 1846)	15	-	b
	3	*Ctenactis echinata* (Pallas, 1766)	12	-	a
	4	*Cycloseris boschmai* Hoeksema, 2014	6	2b	-
	5	*Cycloseris costulata* (Ortmann, 1889)	12	2c	-
	6	*Cycloseris cyclolites* (Lamarck, 1815)	1	-	-
	7	*Cycloseris explanulata* (Van der Horst, 1922)	2	2d	-
	8	*Cycloseris mokai* (Hoeksema, 1989)	6	2e	-
	9	*Cycloseris sinensis* Milne Edwards & Haime, 1851	1	-	-
	10	*Cycloseris tenuis* (Dana, 1846)	11	2f	-
	11	*Danafungia horrida* (Dana, 1846)	13	2g	a
	12	*Danafungia scruposa* (Klunzinger, 1879)	13	2h	a
	13	*Fungia fungites* (Linnaeus, 1758)	15	3a	a, b
	14	*Halomitra pileus* (Linnaeus, 1758)	5	3b	a
	15	*Herpolitha limax* (Esper, 1797)	15	3c	a, b
	16	*Lithophyllon concinna* (Verrill, 1864)	13	-	-
	17	*Lithophyllon ranjithi* Ditlev, 2003	7	3d	-
	18	*Lithophyllon repanda* (Dana, 1846)	17	3e	b
	19	*Lithophyllon scabra* (Döderlein, 1901)	12	3f	-
	20	*Lithophyllon undulatum* Rehberg, 1892	4	3g	a
	21	*Lobactis scutaria* (Lamarck, 1801)	15	3h	b
	22	*Pleuractis granulosa* (Klunzinger, 1879)	12	4a	-
	23	*Pleuractis gravis* (Nemenzo, 1955)	5	4b	-
	24	*Pleuractis moluccensis* (Van der Horst, 1919)	6	4c	a
	25	*Pleuractis paumotensis* (Stutchbury, 1833)	16	-	-
	26	*Podabacia motuporensis* Veron, 1990	1	-	-
	27	*Podabacia sinai* Veron, 2000	1	4d	-
	28	*Polyphyllia talpina* (Lamarck, 1801)	1	4e	a, b
	29	*Sandalolitha boucheti* Hoeksema, 2012	2	4f	-
	30	*Sandalolitha dentata* Quelch, 1884	12	4g	-
	31	*Sandalolitha robusta* (Quelch, 1886)	9	4h	a, b
Agariciidae	32	*Coeloseris mayeri* Vaughan, 1918*	3	5a	-
	33	*Gardineroseris planulata* (Dana, 1846)	7	5b	a
	34	*Leptoseris foliosa* Dinesen, 1980	5	5c	-
	35	*Leptoseris glabra* Dinesen, 1980	17	5d	-
	36	*Leptoseris hawaiiensis* Vaughan, 1907	12	5e	-
	37	*Leptoseris incrustans* (Quelch, 1886)	9	5f	-
	38	*Leptoseris kalayaanensis* Licuanan and Aliño, 2009	13	5g	-
	39	*Leptoseris mycetoseroides* Wells, 1954	18	5h	a
	40	*Leptoseris scabra* Vaughan, 1907	12	6a	-
	41	*Leptoseris solida* (Quelch, 1886)	6	6b	-
	42	*Leptoseris troglodyta* Hoeksema, 2012	1	6c	-
	43	*Leptoseris yabei* (Pillai and Sheer, 1976)	1	6d	-
	44	*Pachyseris rugosa* (Lamarck, 1801)*	8	6e	a
	45	*Pacyhseris speciosa* (Dana, 1846)*	6	6f	a
	46	*Pavona bipartita* Nemenzo, 1980	3	6g	-
	47	*Pavona clavus* (Dana, 1846)	3	6h	a
	48	*Pavona duerdeni* Vaughan, 1907	5	7a	-
	49	*Pavona explanulata* (Lamarck, 1816)	6	7b	a
	50	*Pavona maldivensis* (Gardiner, 1905)	4	7c	-
	51	*Pavona minuta* Wells, 1954	6	7d	a
	52	*Pavona varians* Verrill, 1864	14	7e	a
	53	*Pavona venosa* (Ehrenberg, 1834)	9	7f	-
Euphylliidae	54	*Euphyllia glabrescens* (Chamisso & Eysenhardt, 1821)	1	8a	a
	55	*Euphyllia paraancora* Veron, 1990	1	8b	-
	56	*Physogyra lichtensteini* Milne Edwards & Haime, 1851*	1	8c	a

Other coral species recorded at Layang-Layang during earlier studies, but not encountered during the present survey are the fungiids *Heliofungia actiniformis* (Quoy & Gaimard, 1833) and *Podabacia crustacea* (Pallas, 1766), the agariciids *Pavona cactus* (Forskål, 1775) and *Pavona decussata* (Dana, 1846), and the euphylliids *Euphyllia ancora* Veron & Pichon, 1980 and *Plerogyra sinuosa* (Dana, 1846) (Pilcher et al. 1999, Pilcher and Cabanban 2000). The presence of *Heliofungia actiniformis* and both euphylliids was verified by images in Pilcher et al. (1999), thus bringing the total species count to 59 (Table 4).

**Table 4.** Hard coral species that were not encountered in the present study. Species marked with an asterisk (*) is now considered *incertae sedis*. Previous records are indicated as follows: a = Pilcher et al. (1999), b = Pilcher and Cabanban (2000). The presence of *Heliofungia actiniformis*, *Euphyllia ancora* and *Plerogyra sinuosa* are verified by images in Pilcher et al. (1999).

Family	No.	Species	Previous records	Status
Fungiidae	1	*Heliofungia actiniformis* (Quoy & Gaimard, 1833)	a, b	Verified
	2	*Podabacia crustacea* (Pallas, 1766)	b	Unverified
Agariciidae	3	*Pavona cactus* (Forskål, 1775)	b	Unverified
	4	*Pavona decussata* (Dana, 1846)	b	Unverified
Euphylliidae	5	*Euphyllia ancora* Veron & Pichon, 1980	a, b	Verified
	6	*Plerogyra sinuosa* (Dana, 1846)*	a, b	Verified

### *Pavona* corals – molecular perspective

**Figure 10.**
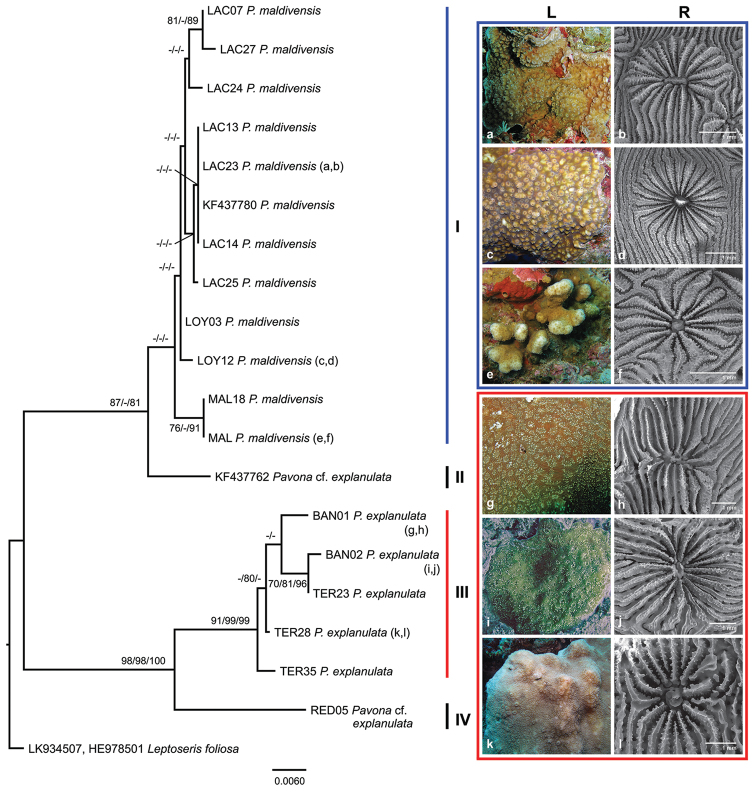
Maximum likelihood phylogram of *Pavona maldivensis* and *Pavona explanulata* based on combined mitochondrial intergenic spacer between CO1 and 16S-rRna and nuclear marker ITS1-5.8S-ITS2 sequences. Support values for maximum likelihood, maximum parsimony (>70) and bayesian posterior probabilities (>80) are given at the nodes. Dashes (-) indicate nodes without statistical support. Letters in parentheses correspond to images a–l in rows L and R. L: coral colonies *in situ*, R: SEM images of calices. Locality for the coral samples are: LAC = Layang-Layang, LOY = Loyalty Islands, MAL = the Maldives, BAN = Banggi, East Malaysia, TER = Ternate, Indonesia, RED = Redang, Peninsular Malaysia. **a–b** *Pavona maldivensis* from Layang-Layang **c–d** *Pavona maldivensis* from Loyalty Islands **e–f** *Pavona maldivensis* from the Maldives **g–h** *Pavona explanulata* from Banggi, Sabah **i–j** *Pavona explanulata* from Banggi, Sabah **k–l** *Pavona explanulata* from Ternate, Indonesia. Additional samples KF437780 *Pavona maldivensis* and KF437762 Pavona cf. explanulata are from Pearl and Hermes Atoll, the northwest Hawaiian Islands and O’ahu, Hawaii, respectively (Luck et al. 2013). Outgroup LK934507, HE978501 *Leptoseris foliosa* is from Prony Bay, New Caledonia (Benzoni et al. 2012b, Terraneo et al. 2014).

Sequences were obtained from 11 and 17 *Pavona* samples for the IGR and ITS markers, respectively. Amplification success for the IGR marker was rather low and the length of the sequences ranged between 432 and 887 bp, shorter than the expected length of ~1200 bp (Terraneo et al. 2014). Tree topologies obtained from the ML, MP and BI analyses for each gene and the concatenated dataset were comparable so only the ML phylogram is shown. There were some differences in the topology between the IGR and ITS phylogeny trees, but the ITS tree was less resolved and has lower support values (Suppl. material 4). The topology of the IGR tree is almost similar with the concatenated sequences tree and has well-supported basal clades, hence we focus on the latter. The final alignment of the concatenated sequences consisted of 1360 characters with 1243 constant, 35 variable and 82 parsimony informative characters. The phylogram consists of four clades (Figure 10). Samples of the *Pavona maldivensis* from Layang-Layang (samples with LAC labels) clustered with those of *Pavona maldivensis* from other areas (clade I). Clade II consists of a single specimen of Pavona cf. explanulata from Hawaii (Luck et al. 2013). Samples BAN02 from Banggi, North Borneo and TER28 from Ternate, Indonesia, initially identified as *Pavona maldivensis* during in situ observations from previous studies clustered together with *Pavona explanulata* samples (clade III). Pavona cf. explanulata from Redang, Peninsular Malaysia formed clade IV. The pairwise genetic difference between clades, although considerably low, were highest between clade II and clade IV (0.073 ± 0.011), followed by clade II and clade III (0.066 ± 0.011) and clade I and clade IV (0.047 ± 0.005). The intraspecific genetic distance within the clades was also very low: 0.004 ± 0.001 for clade I and 0.008 ± 0.002 for clade III.

### *Pavona* corals – morphology

Macro- and micromorphology features of the *Pavona* corals support the clades of the molecular analyses (Figure 10a–l, Suppl. material 5). In general, the *Pavona maldivensis* specimens from Layang-Layang were small in size, the largest measured 11 cm × 8 cm while the smallest was 3.5 cm × 3 cm. All *Pavona maldivensis* specimens from Layang-Layang had paper-thin coralla (≤1 mm) and were found encrusting the reef wall (e.g. Figure 7c). Several specimens had knobs or rounded columns protruding from the corallum (Figure 10a). The specimen from the Maldives has a columnar or club-shaped growth form (Figure 10e). The corallite morphology is variable within the same specimen, largely depending on the position of the corallites in the corallum. Calices at the top of the knobs or columns are small and compact and become larger and widely spaced towards the base or on horizontal plates (see Gardiner 1905). Calices vary from circular, distinctly raised edges (plocoid) (Figure 10d) to broad, flattened edges particularly at the base of the colony (Figure 10f), or a combination of both features (Figure 10b) when inclined towards the margin. Calices with raised walls protrude up to 2–4 mm. The columella is well-developed in the form of a peg (Figure 10b) or a single, rounded or twisted rod (Figure 10d, f).

**Figure 11.**
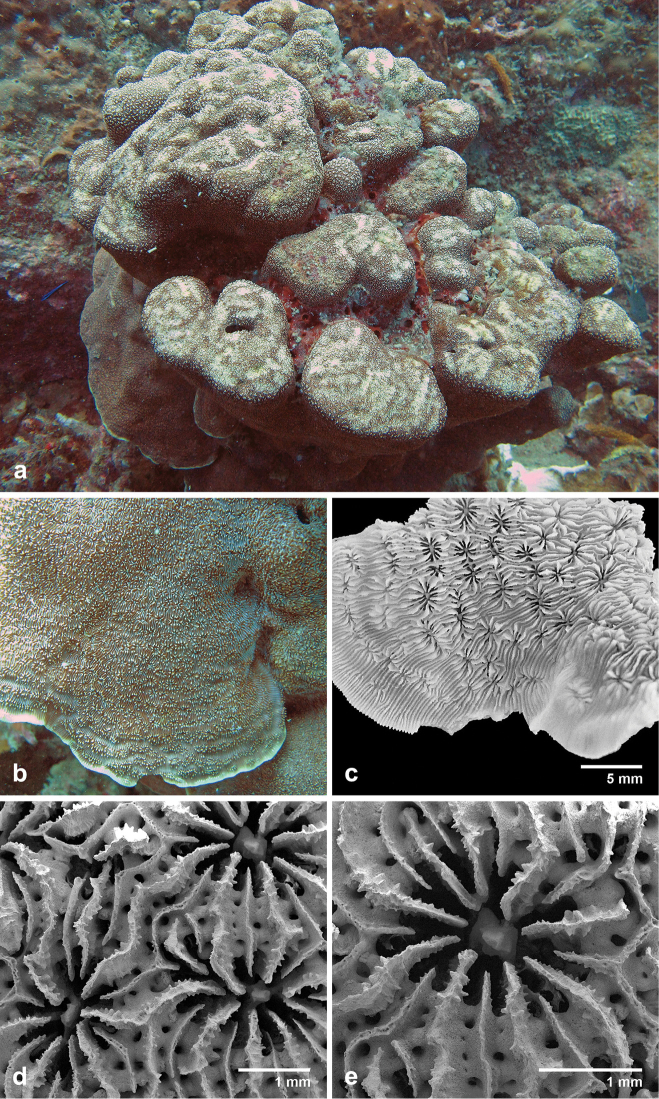
Specimen RED05 Pavona cf. explanulata from Pulau Redang, Peninsular Malaysia. **a** coral colony *in situ* at 13 m depth **b** corallites detail at the plate margin **c** coral fragment preserved in ethanol **d, e** SEM images showing calices.

Specimens of *Pavona explanulata* were either encrusting (Figure 10g, i), submassive (Figure 10k) or a combination of submassive with plate margins. Corallites may have irregular arrangements and shapes and mostly lack any form of wall (theca), giving the corallum a smooth surface appearance. Veron and Pichon (1980) described the thecae as “synapticulothecate”, if present, which is defined as rod-or bar-like structures extending between the septa (Budd et al. 2012). In plate colonies, the corallites are inclined towards the margin and usually in parallel rows. The columella consists of several fused processes that extend from the radial elements into the fossa (Figure 10h) or a single process, which appears as a twisted rod (Figure 10j, l). Synapticular rings may be visible in this species (Figure 10j, l). The specimen from Redang, Peninsular Malaysia looks superficially like *Pavona explanulata*, but the morphology differs from the rest of the *Pavona explanulata* specimens by the deeply seated columella and the widely spaced septa (Figure 11). This specimen resembles Pavona cf. explanulata in Veron and Pichon (1980: Fig. 31).

**Figure 12.**
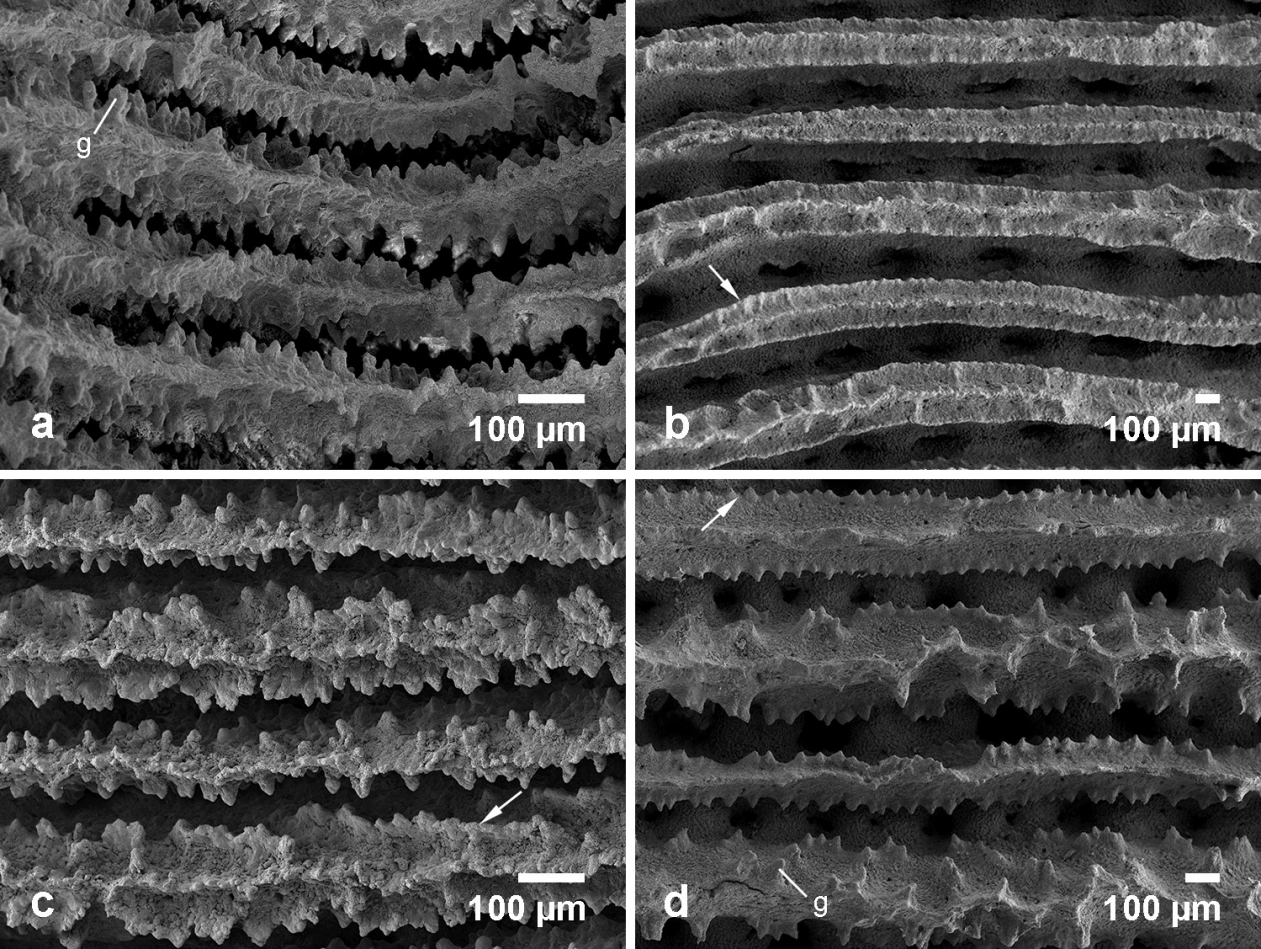
Variation of radial elements in *Pavona maldivensis* (**a**, **c**) and *Pavona explanulata* (**b, d**). **a** specimen LAC23 from Layang-Layang, **b** specimen BAN02 from Banggi, North Borneo, **c** specimen LOY12 from Loyalty Islands, and **d** specimen TER28 from Ternate, Indonesia. Lateral faces of septa are covered with granules (g) as indicated in **a** and **d**. Aligned granulations along the lateral faces of septa (menianae) are indicated by white arrows in **b, c, d**.

Septocostae of both *Pavona* species are closely compacted, but in *Pavona maldivensis* they have denser granulated sides (Figure 12). In *Pavona explanulata*, the order of septa may alternate between thin with rows of fine granules and thick with prominent spines. The upper margin of the septa (forming radial elements) consist of beaded granules in *Pavona maldivensis*, and in *Pavona explanulata* it appears to taper into a somewhat straight ridge. The side walls or lateral faces of the septa are covered with granules either in rows or scattered on the surface (e.g. Figure 12a, d). Aligned granulations alongside the lateral faces also known as menianae (Kitahara et al. 2010, Benzoni et al. 2012b, Terraneo et al. 2014), or menianes (Kitahara et al. 2012, Hoeksema 2012b), are more obvious in *Pavona explanulata* (Figure 12b, d) as compared with *Pavona maldivensis*, which has short series of menianae, if formed (Figure 12c). Radial elements of *Pavona explanulata* can have almost smooth margins (e.g. Figure 12b), and this has been described by Veron and Pichon (1980) for the second order septa of this species. A summary of the variation in morphological characters between *Pavona maldivensis* and *Pavona explanulata* is given in Table 5.

**Table 5.** Summary of variation in morphological characters between *Pavona maldivensis* and *Pavona explanulata*.

Morphological characters	*Pavona maldivensis*	*Pavona explanulata*
Growth form	Encrusting; paper-thin coralla, club-shaped (specimen from the Maldives)	Encrusting, submassive; may have plate margins
Corallite (arrangement)	Small and compact (top of the colony), widely spaced (base of the colony)	Irregular spacing and arrangement, in parallel rows towards the margin
Corallite (shape)	Circular, plocoid, flattened edges when inclined towards the margin	Irregular shape, but may be somewhat circular
Theca	Distinct; plocoid	Mostly lacking, synapticulothecate if present
Columella	Well-developed; peg-like, single, rounded or twisted rod	Several fused processes
Septo-costae	Compact, dense granulated sides	Compact, septa alternate between thin with fine granules and thick with spines
Radial elements	Beaded granules	Tapers into straight ridge, may have smooth margins
Lateral faces	Granules in rows or scattered on the surface, may form short series of menianae	With granules and meaninae

## Discussion

### Species occurrence

The total number of coral species noted at Layang-Layang is lower than that of other localities in Sabah where similar surveys have been conducted. Nevertheless, the overall species diversity is considered high even for fungiid corals, despite the fact that steep reef walls are hostile environments for free-living mushroom corals, as they can get dislodged from these drop-offs (Hoeksema and Moka 1989). In contrast, agariciid corals of the genus *Leptoseris* are known to flourish at greater depths (Kahng and Maragos 2006, Rooney et al. 2010, Dinesen et al. 2012, Luck et al. 2013). Based on the three target coral families, there is a decrease in the number of species from the east to the west coast of Sabah, from Semporna (90 species: 44 Fungiidae, 31 Agariciidae, 15 Euphylliidae; Waheed and Hoeksema 2013) to Kudat (84 species: 39 Fungiidae, 30 Agariciidae, 15 Euphylliidae; unpublished data), then Kota Kinabalu (72 species: 35 Fungiidae, 26 Agariciidae, 11 Euphylliidae; Waheed and Hoeksema 2014), and finally offshore Layang-Layang (59 species: 32 Fungiidae, 22 Agariciidae, 5 Euphylliidae) (see inset of Figure 1). This is concordant with the general pattern of decreasing diversity away from the designated centre of maximum marine biodiversity (Briggs 1974, Hoeksema 2007, 2013, 2015, Bellwood and Meyer 2009), also known as the Coral Triangle, with its present western border at the Sulu Sea (Veron et al. 2009, 2011). However, Huang et al. (2015) demonstrated that there is no significant structure in species diversity on a larger scale from the eastern to the western reefs of the South China Sea, and suggested that local dynamics are at play in driving the species richness and distribution patterns in the area. Based on their coral species composition, the Spratly Islands clustered together with southern Vietnam rather than closer continental localities such as Sabah, Brunei or Palawan, the Philippines (Huang et al. 2015), which implies that the former two localities may have similar reef attributes. For oceanic Layang-Layang with its steep reefs walls, an additional factor for its lower species richness as compared to nearby continental reef areas such as Kota Kinabalu and Kudat could be the lack of habitat heterogeneity, which would be important for supporting species diversity (Done 1982, Best et al. 1989; Cornell and Karlson 1996, 2000, Karlson and Cornell 1998, Hoeksema 2007, 2012c).

Among the new coral records for Layang-Layang are five rarely documented species, i.e., *Lithophyllon ranjithi* (Figure 3d), *Podabacia sinai* (Figure 4d), *Sandalolitha boucheti* (Figure 4f), *Leptoseris kalayaanensis* (Figure 5g) and *Leptoseris troglodyta* (Figure 6c). *Lithophyllon ranjithi*, a mushroom coral previously thought to be endemic to northeast Borneo was encountered at seven sites. The first mention of this species from the South China Sea was from Brunei (Turak and DeVantier 2011), although this record could not be confirmed in a later review (Hoeksema and Lane 2014). This species is usually found on nearshore or coastal reefs (Waheed and Hoeksema 2013) so it was surprising to find it at an offshore and oceanic locality. Conversely, *Leptoseris kalayaanensis* was anticipated to be present in Layang-Layang. In fact, its type locality, the Kalayaan Islands in the Philippines is located ~ 350 km northeast from the atoll and this species has been reported from three other localities in the South China Sea, two with rather similar reef conditions (Licuanan and Aliño 2009) and one from the coastal area of Nha Trang, Vietnam (Hoeksema et al. 2010). Specimens of *Leptoseris kalayaanensis* were found at 13 sites along steep reef walls and considered rather common in Layang-Layang. *Leptoseris troglodyta*, a cavernicolous and azooxanthellate species was encountered only once during the dives. A cluster of about 12 small calices was found on the ceiling of a small cave in a reef wall at 30 m depth (Site 1). This species is widely distributed in the West Pacific (Hoeksema 2012b) and this account is the first record for the South China Sea. *Podabacia sinai* was previously recorded from the Red Sea (Veron 2000) but meanwhile it has also been found in various other Indo-Pacific localities (Hoeksema unpubl.). *Sandalolitha boucheti* has been observed in Vanuatu (Hoeksema 2012a) and Semporna, East Sabah (Waheed and Hoeksema 2013). This species was represented on the Layang-Layang reefs by two specimens. The largest of these (Ø ~ 6 cm; Figure 4f) was still attached by a very wide stalk while the other was free-living and showed a large detachment scar. It is abnormal for free-living mushroom corals to maintain a long-lasting fixed growth form (Hoeksema and Yeemin 2011), which may hinder their identification.

Corals of three *Leptoseris* spp. could not be identified to species level. Two specimens of encrusting *Leptoseris* sp. 1 had free margins and small corallites (≤ 1.5 mm in size). One of these corals (Figure 9a) has calices in rows somewhat concentric and parallel with the margin and a central corallite can be discerned. Corallites appear sunken because of the high and continuous carinae. This specimen measures Ø 5 cm, and it is possibly a juvenile *Leptoseris mycetoseroides*. Further examination of these specimens is required for certainty. Unfortunately, specimens of *Leptoseris* sp. 2 and 3 were not collected; nevertheless photos have been included for visual record (Figure 9b, c).

Three coral species that have been reported from Layang-Layang but were not observed in the present study are the fungiid *Podabacia crustacea* and the agariciids *Pavona cactus* and *Pavona decussata* (Pilcher and Cabanban 2000). Since there is no photographic evidence or other supporting information to support the presence of these species on the atoll, they have not been included in the current species list. Still, it is very likely that these three species are present on the reef as they are common Indo-Pacific species and their distribution ranges certainly cover across the South China Sea, including the Spratly Islands (see Huang et al. 2015). As a note, *Pavona cactus* is usually found in turbid and sheltered reef conditions (Veron and Pichon 1980, Veron 2000). However, *Pavona cactus* and *Pavona decussata* can also be found in shallow reef environments such as upper reef slopes and lagoons. It is possible that these species are present in the lagoon reefs, which were underexplored in our study, as surveys were only possible around the reef walls of the atoll.

Many coral colonies in Layang-Layang appeared small in size, and most were juveniles. This was consistent across the reef sites for most coral families including those targeted in our study. For example, the largest collected *Leptoseris kalayaanensis* specimen measured 7.5 cm x 5 cm while the smallest was 4 cm × 3 cm. Also, the initial uncertainty in identifying *Pavona maldivensis* most likely stemmed from the fact that the specimens were very small in size (collected colonies were between 11 cm × 8 cm and 3.5 cm × 3 cm). This puzzling find can be explained by the fact that corals in the study area are recolonizing after the outbreak of the corallivorous crown-of-thorns (COT) seastar. Surveys during the COT outbreak in July 2010 were conducted down to 10 m depth only (Nasrulhakim et al. 2010), but the damage extends deeper based on our surveys (~ 20 m depth). Preceding this event there was also a COT outbreak on the nearby reefs of Brunei in April-May 2010, and at the same time a report of large numbers of COT at the reefs of the Tunku Abdul Rahman Park in Kota Kinabalu, Sabah on the northwest coast of Borneo (Lane 2012). Following this outbreak, Brunei and Kota Kinabalu reported to have thermally induced bleaching episodes in June-July 2010 (Lane 2011, Aw and Muhammad Ali 2012). There is no account on whether the reefs of Layang-Layang were also affected by this bleaching event, and based on our observations, we are unable to determine if a bleaching episode did occur.

There is no information on the extent of damage caused to the reefs and the rate of recovery from the COT outbreak in 2010, as the reefs of Layang-Layang are not monitored regularly. Many studies have reported the effects of COTs to reefs in terms of coral cover loss and changes in coral assemblage (e.g. Lourey et al. 2000, Pratchett 2010, Lane 2011, 2012, Baird et al. 2013, Bos et al. 2013, Osborne et al. 2011, Saponari et al. 2014). While COTs are known to have a feeding preference for *Acropora* (De’ath and Moran 1998, Pratchett 2007), they have also been found to feed on many different coral taxa (Glynn 1974, Ormond et al. 1976, Colgan 1987, De’ath and Moran 1998, Pratchett 2007, Pratchett et al. 2009), which includes fungiids (De’ath and Moran 1998, Pratchett 2007, 2010, Pratchett et al. 2009, Scott et al. 2015) and agariciids (Colgan 1987, Pratchett 2007, 2009, 2010) particularly during an outbreak or when food becomes scarce (Moran 1986). Similarly for Layang-Layang, it appears that the COT outbreak resulted in high coral mortality (E Foo, J Bell, R Wahab, Avillon Layang-Layang Resort, pers. comm.). Our study was carried out almost three years after the outbreak and from our observations, the high frequency of coral recruits and small-sized colonies indicate an ongoing recovery of corals. We question whether all coral species (as prior to the outbreak) have re-established on the reefs as coral species such as *Euphyllia ancora* and *Plerogyra sinuosa*, both reported as common from previous surveys (NJ Pilcher, pers. comm.), were not observed during the present study. We are unable to draw a conclusion that the absence of certain species previously reported for Layang-Layang is caused by the 2010 COT event. While some baseline data is available for comparison (e.g. Pilcher et al. 1999, Pilcher and Cabanban 2000), there is no specific locality data of species occurrences to refer to. As far as we know, the reefs of Layang-Layang were monitored between 1996 and 1999 (see Pilcher et al. 1999, Pilcher and Cabanban 2000) and since then and prior to that, reef surveys were conducted intermittently. As several studies have impressed upon the importance of utilising long-term monitoring data in order to assess changes to the coral communities (e.g. Brown et al. 2002, Somerfield et al. 2008), there is a need to establish a coral reef monitoring plan for Layang-Layang. With a monitoring plan in place, any changes or disturbance can be detected at the onset so that mitigation measures can be taken if necessary.

### *Pavona* species boundaries

The IGR marker has proven to be successful in resolving species boundaries in the family Agariciidae and the genus *Pachyseris* (Terraneo et al. 2014). In our small dataset, the IGR marker gave better resolution than the ITS marker in resolving species-level relationships for two *Pavona* species. Both the IGR and the concatenated gene tree supported two main groups, one of *Pavona maldivensis*, including specimens from Layang-Layang, and the other of *Pavona explanulata*.

For all specimens in the *Pavona maldivensis* clade, the calice size is smaller, the calice walls are raised and distinct, the septocostae spacing is more compact and there is more surface ornamentation on the radial elements as compared to *Pavona explanulata*. They share some similar features in colony growth form, but *Pavona explanulata* does not form club-shaped branches. *Pavona explanulata* specimens also tend to have a smooth surface appearance due to the absent calice wall, but exceptions do occur. The columella is well-developed as a single, rounded or twisted rod for both species, peg-like for *Pavona maldivensis* and as fused processes in *Pavona explanulata*. Lastly, synapticular rings are obvious in *Pavona explanulata* but not so in *Pavona maldivensis*, though Veron and Pichon (1980) have described them to be obvious on the branch ends.

Upon re-examining the morphological characters of the *Pavona* specimens identified as *Pavona maldivensis* in our previous studies (Waheed and Hoeksema 2013, 2014), it was clear that the specimens were more similar to *Pavona explanulata* instead. However, these specimens have calices with somewhat distinct walls, a feature that is more typical of *Pavona maldivensis*. This is most likely one of the factors that prompted the misidentification of these specimens. Adding to this, *Pavona maldivensis* has a wide distribution range in the Indo-Pacific (e.g. Veron and Pichon 1980, Scheer and Pillai 1983, Maragos and Jokiel 1986, Dai and Lin 1992, Nishihira and Veron 1995, Glynn et al. 2007, Pichon 2007, Pichon and Benzoni 2007), and has been reported from the Bodgaya and Sipadan islands in Semporna, Sabah (Wood and Tan 1987); hence, we had expected to find this species in our previous study areas (i.e. Semporna and Kota Kinabalu).

Although *Pavona maldivensis* and *Pavona explanulata* may not be considered the most problematic species within *Pavona*, specimens that closely resemble these species have been collected and analysed. For example, sample KF437762 Pavona cf. explanulata (Luck et al. 2013) clusters basally to *Pavona maldivensis* rather than with its conspecifics, while sample RED05 Pavona cf. explanulata from Redang clusters basally to the other samples of *Pavona explanulata*. For the latter, the macro- and micromorphology of this specimen was noticeably different from the rest of the *Pavona explanulata* samples. These cases indicate that the identity of *Pavona explanulata* should be carefully re-examined in the future through a larger morpho-molecular study including several specimens from various localities.

### Implication of misidentified *Pavona maldivensis*

Based on the findings of this study, the “true” *Pavona maldivensis* has only been found in Layang-Layang out of the other localities previously visited in Sabah, Malaysia, i.e. Semporna and Kota Kinabalu (Waheed and Hoeksema 2013, 2014), and the status of this species in those localities remains ambiguous until future data is available. It is highly likely that the misidentified specimens from those previous studies are *Pavona explanulata*, as was discovered for specimens from Banggi and Ternate utilised in this study, or a variety closely resembling it.

The name *Pavona explanulata*, like *Pavona maldivensis*, has been mistakenly used in the past (examples given by Veron and Pichon 1980: 17–36). Furthermore, the type specimen of *Pavona explanulata* appears to be missing and the original species description is rather vague, so taxonomic literature of this species since when it was first described needs to be re-examined in order to better define its species boundaries.

## Conclusions

The coral species list for the families Fungiidae, Agariciidae and Euphylliidae in the present study added 32 new records for Layang-Layang and includes rarely recorded species such as *Leptoseris kalayaanensis*, which is thus far a South China Sea endemic. The mushroom coral *Lithophyllon ranjithi* has a wider distribution range than previously thought and can no longer be considered endemic to northeastern Borneo. This is the first record of this species from an oceanic and offshore reef habitat, in contrast to its previously reported habitat preference for coastal and sheltered reef conditions.

An integrative molecular and morphological approach was utilised to determine that specimens identified as *Pavona maldivensis* from previous surveys are in fact *Pavona explanulata*. The combination of both techniques have proven to be powerful in addressing species complexes in scleractinians (e.g. Benzoni et al. 2007, 2011, 2014, Kitahara et al. 2012, Arrigoni et al. 2014a, b, c, Kitano et al. 2014), particularly if type specimens and coral samples from the type locality are included in the analyses (Huang et al. 2014), and taxonomic descriptions are consolidated (Benzoni et al. 2010). While the species boundaries between *Pavona maldivensis* and *Pavona explanulata* may already be distinct based on morphological descriptions and images in current taxonomic literature, the present study has included SEM images of calices and radial elements of specimens of both species for the first time to further illustrate the previous descriptions. In addition, a specimen closely resembling but dissimilar from *Pavona explanulata* was also shown. As such, this finding may serve as a stepping stone for further investigations of *Pavona*.

## Supplementary materials

Supplementary material 1 Table S1 Data type: measurement Explanation note: Physical properties of seawater at Pulau Layang-Layang. This dataset is made available under the Open Database License (http://opendatacommons.org/licenses/odbl/1.0/). The Open Database License (ODbL) is a license agreement intended to allow users to freely share, modify, and use this Dataset while maintaining this same freedom for others, provided that the original source and author(s) are credited.Zarinah Waheed, Francesca Benzoni, Sancia E.T. van der Meij, Tullia I. Terraneo, Bert W. Hoeksema

Supplementary material 2 Table S2 Data type: specimens data Explanation note: Coral samples included in the molecular analyses with supporting collection and locality data. This dataset is made available under the Open Database License (http://opendatacommons.org/licenses/odbl/1.0/). The Open Database License (ODbL) is a license agreement intended to allow users to freely share, modify, and use this Dataset while maintaining this same freedom for others, provided that the original source and author(s) are credited.Zarinah Waheed, Francesca Benzoni, Sancia E.T. van der Meij, Tullia I. Terraneo, Bert W. Hoeksema

Supplementary material 3 Table S3 Data type: specimens data Explanation note: Species occurrence of hard coral families Fungiidae, Agariciidae and Euphylliidae at Pulau Layang-Layang. This dataset is made available under the Open Database License (http://opendatacommons.org/licenses/odbl/1.0/). The Open Database License (ODbL) is a license agreement intended to allow users to freely share, modify, and use this Dataset while maintaining this same freedom for others, provided that the original source and author(s) are credited.Zarinah Waheed, Francesca Benzoni, Sancia E.T. van der Meij, Tullia I. Terraneo, Bert W. Hoeksema

Supplementary material 4 Figure S1 Data type: molecular data Explanation note: Maximum likelihood phylogram of *Pavona maldivensis* and *Pavona explanulata*. **a** mitochondrial intergenic spacer between CO1 and 16S-rRna. The sequence alignment consisted of 680 characters with 593 constant, 23 variable and 64 parsimony informative characters, **b** nuclear marker ITS1-5.8S-ITS2, with 680 characters of 649 constant, 14 variable and 17 parsimony informative characters. Support values for maximum likelihood, maximum parsimony (>70) and bayesian posterior probabilities (>80) are given at the nodes. Dashes (-) indicate nodes without statistical support. This dataset is made available under the Open Database License (http://opendatacommons.org/licenses/odbl/1.0/). The Open Database License (ODbL) is a license agreement intended to allow users to freely share, modify, and use this Dataset while maintaining this same freedom for others, provided that the original source and author(s) are credited.Zarinah Waheed, Francesca Benzoni, Sancia E.T. van der Meij, Tullia I. Terraneo, Bert W. Hoeksema

Supplementary material 5 Figure S2 Data type: specimens data Explanation note: Variation of corallite form and structure in *Pavona maldivensis* (**a**, **c**, **e**), and *Pavona explanulata* (**b**, **d**, **f**). **a** specimen LAC23 from Layang-Layang, **b** specimen BAN01 from Banggi, North Borneo, **c** specimen LOY12 from Loyalty Islands, **d** specimen BAN02 from Banggi, North Borneo, **e** specimen MAL from the Maldives, and **f** specimen TER28 from Ternate, Indonesia. This dataset is made available under the Open Database License (http://opendatacommons.org/licenses/odbl/1.0/). The Open Database License (ODbL) is a license agreement intended to allow users to freely share, modify, and use this Dataset while maintaining this same freedom for others, provided that the original source and author(s) are credited.Zarinah Waheed, Francesca Benzoni, Sancia E.T. van der Meij, Tullia I. Terraneo, Bert W. Hoeksema

## References

[B1] Abdullah MP (2005) Marine Research Station Layang-Layang Malaysia (MARSAL) 2004 & 2005 highlights. Department of Fisheries Malaysia and Ministry of Agriculture and Agro-based Industry, 52 pp http://www.fri.gov.my/marsal/publications.html

[B2] Akhir MFM (2012) Surface circulation and temperature distribution of southern South China Sea from Global Ocean Model (OCCAM). Sains Malaysiana 41: 701–714.

[B3] Arrigoni R Terraneo TI Galli P Benzoni F (2014a) Lobophylliidae (Cnidaria, Scleractinia) reshuffled: Pervasive non-monophyly at genus level. Molecular Phylogenetics and Evolution 73: 60–64. doi: 10.1016/j.ympev.2014.01.010 24472672 10.1016/j.ympev.2014.01.010

[B4] Arrigoni R Kitano YF Stolarski J Hoeksema BW Fukami H Stefani F Galli P Montano S Castoldi E Benzoni F (2014b) A phylogeny reconstruction of the Dendrophylliidae (Cnidaria, Scleractinia) based on molecular and micromorphological criteria, and its ecological implications. Zoologica Scripta 43: 661–688. doi: 10.1111/zsc.12072

[B5] Arrigoni R Richards ZT Chen CA Baird AH Benzoni F (2014c) Taxonomy and phylogenetic relationships of the coral genera *Australomussa* and *Parascolymia* (Scleractinia, Lobophylliidae). Contributions to Zoology 83: 195–215.

[B6] Aw SL Muhammad Ali SY (2012) Coral bleaching event in Kota Kinabalu, Sabah, Malaysia. In: International Seminar on Marine Science and Aquaculture, 11–13 March 2012 Universiti Malaysia Sabah, Kota Kinabalu, 80.

[B7] Baird AH Pratchett MS Hoey AS Herdiana Y Campbell SJ (2013) *Acanthaster planci* is a major cause of coral mortality in Indonesia. Coral Reefs 32: 803–812. doi: 10.1007/s00338-013-1025-1

[B8] Bellwood DR Meyer CP (2009) Searching for heat in a marine biodiversity hotspot. Journal of Biogeography 36: 569–576. doi: 10.1111/j.1365-2699.2008.02029.x

[B9] Benzoni F Stefani F Stolarski J Pichon M Mitta G Galli P (2007) Debating phylogenetic relationships of the scleractinian *Psammocora*: molecular and morphological evidences. Contributions to Zoology 76: 35–54.

[B10] Benzoni F Stefani F Pichon M Galli P (2010) The name game: morpho‐molecular species boundaries in the genus *Psammocora* (Cnidaria, Scleractinia). Zoological Journal of the Linnean Society 160: 421–456. doi: 10.1111/j.1096-3642.2010.00622.x

[B11] Benzoni F Arrigoni R Stefani F Pichon M (2011) Phylogeny of the coral genus *Plesiastrea* (Cnidaria, Scleractinia). Contributions to Zoology 80: 231–249.

[B12] Benzoni F Arrigoni R Stefani F Reijnen BT Montano S Hoeksema BW (2012a) Phylogenetic position and taxonomy of *Cycloseris explanulata* and *C. wellsi* (Scleractinia: Fungiidae): lost mushroom corals find their way home. Contributions to Zoology 81: 125–146.

[B13] Benzoni F Arrigoni R Stefani F Stolarski J (2012b) Systematics of the coral genus *Craterastrea* (Cnidaria, Anthozoa, Scleractinia) and description of a new family through combined morphological and molecular analyses. Systematic and Biodiversity 10: 417–433. doi: 10.1080/14772000.2012.744369

[B14] Benzoni F Arrigoni R Waheed Z Stefani F Hoeksema BW (2014) Phylogenetic relationships and revision of the genus *Blastomussa* (Cnidaria: Anthozoa: Scleractinia) with description of a new species. Raffles Bulletin of Zoology 62: 358–378.

[B15] Best MB Hoeksema BW Moka W Moll H Sutarna IN (1989) Recent scleractinian coral species collected during the Snellius-II Expedition in eastern Indonesia. Netherlands Journal of Sea Research 23: 107–115. doi: 10.1016/0077-7579(89)90005-7

[B16] Bos AR Gumanao GS Mueller B Saceda-Cardoza MM (2013) Management of crown-of-thorns sea star (*Acanthaster planci* L.) outbreaks: removal success depends on reef topography and timing within the reproduction cycle. Ocean and Coastal Management 71: 116–122. doi: 10.1016/j.ocecoaman.2012.09.011

[B17] Briggs JC (1974) Marine Zoogeography. McGraw-Hill, New York, 475 pp.

[B18] Brown BE Clarke KR Warwick RM (2002) Serial patterns of biodiversity change in corals across shallow reef flats in Ko Phuket, Thailand, due to the effects of local (sedimentation) and regional (climatic) perturbations. Marine Biology 141: 24–29. doi: 10.1007/s00227-002-0810-0

[B19] Budd AF Fukami H Smith ND Knowlton N (2012) Taxonomic classification of the reef coral family Mussidae (Cnidaria: Anthozoa: Scleractinia). Zoological Journal of the Linnean Society 166: 465–529. doi: 10.1111/j.1096-3642.2012.00855.x

[B20] Colgan MW (1987) Coral reef recovery on Guam (Micronesia) after catastrophic predation by *Acanthaster planci*. Ecology 68: 1592–1605. doi: 10.2307/1939851 10.2307/1939851 29357159

[B21] Cornell HV Karlson RH (1996) Species richness of reef-building corals determined by local and regional processes. Journal of Animal Ecology 65: 233–241. doi: 10.2307/5726

[B22] Cornell HV Karlson RH (2000) Coral species richness: ecological versus biogeographical influences. Coral Reefs 19: 37–49. doi: 10.1007/s003380050224

[B23] Dai CF Lin CH (1992) Scleractinia of Taiwan. Part III. Family Agariciidae. Acta Oceanographica Taiwanica 28: 80–101.

[B24] Darriba D Taboada GL Doallo R Posada D (2012) jModelTest 2: more models, new heuristics and parallel computing. Nature Methods 9: 772. doi: 10.1038/nmeth.2109 10.1038/nmeth.2109 PMC4594756 22847109

[B25] De’ath G Moran PJ (1998) Factors affecting the behaviour of crown of-thorns starfish (*Acanthaster planci* L.) on the Great Barrier Reef: 2: Feeding preferences. Journal of Experimental Marine Biology and Ecology 220: 107–126. doi: 10.1016/S0022-0981(97)00100-7

[B26] Dinesen ZD (1980) A revision of the coral genus *Leptoseris* (Scleractinia: Fungina: Agariciidae). Memoirs of the Queensland Museum 20: 182–235.

[B27] Dinesen ZD Bridge TCL Luck DG Kahng SE Bongaerts P (2012) Importance of the coral genus *Leptoseris* to mesophotic coral communities in the lndo-Pacific. Poster 12th International Coral Reef Symposium, Cairns, 2012 : P101. http://www.icrs2012.com/eposters/P101.pdf

[B28] Ditlev H (2003) New scleractinian corals (Cnidaria: Anthozoa) from Sabah, North Borneo. Description of one new genus and eight new species, with notes on their taxonomy and ecology. Zoologische Mededelingen Leiden 77: 193–219.

[B29] Done TJ (1982) Patterns in the distribution of coral communities across the Central Great Barrier Reef. Coral Reefs 1: 95–107. doi: 10.1007/BF00301691

[B30] Fukami H Chen CA Budd AF Collins A Wallace C Chuang YY Chen C Dai CF Iwao K Sheppard C Knowlton N (2008) Mitochondrial and nuclear genes suggest that stony corals are monophyletic but most families of stony corals are not (order Scleractinia, class Anthozoa, phylum Cnidaria). PLoS ONE 3(9): . doi: 10.1371/journal.pone.0003222 10.1371/journal.pone.0003222 PMC2528942 18795098

[B31] Gardiner JS (1905) Madreporaria III. Fungida IV. Turbinolidae. In: Fauna and geography of the Maldives and Laccadives Archipelagoes, Cambridge 2: 933–957, pls. 89–93.

[B32] Gittenberger A Reijnen BT Hoeksema BW (2011) A molecularly based phylogeny reconstruction of mushroom corals (Scleractinia: Fungiidae) with taxonomic consequences and evolutionary implications for life history traits. Contributions to Zoology 80: 107–132.

[B33] Glynn PW (1974) The impact of *Acanthaster* on corals and coral reefs in the Eastern Pacific. Environmental Conservation 1: 295–303. doi: 10.1017/S037689290000494X

[B34] Glynn PW Wellington GM Riegl B Olson DB Borneman E Wieters EA (2007) Diversity and biogeography of the scleractinian coral fauna of Easter Island (Rapa Nui). Pacific Science 61: 67–90. doi: 10.1353/psc.2007.0005

[B35] Google Earth (2013) Version 7.1.2.2014 https://www.google.com/earth/

[B36] Hall TA (1999) BioEdit: a user-friendly biological sequence alignment editor and analysis program for Windows 95/98/NT. Nucleic Acids Symposium Series 41: 95–98.

[B37] Hancox D Prescott V (1995) A geographical description of the Spratly Islands and an account of hydrographic surveys amongst those islands. Maritime Briefing, Volume 1 Number 6 International Boundaries Research Unit, Department of Geography, University of Durham, Durham, UK, 88 pp.

[B38] Hoeksema BW (1989) Taxonomy, phylogeny and biogeography of mushroom corals (Scleractinia: Fungiidae). Zoologische Verhandelingen, Leiden 254: 1–295.

[B39] Hoeksema BW (2007) Delineation of the Indo-Malayan centre of maximum marine biodiversity. In: Renema W (Ed.) Biogeography, Time, and Place: Distributions, Barriers, and Islands. Springer, Leiden, 117–178. doi: 10.1007/978-1-4020-6374-9_5

[B40] Hoeksema BW (2012a) Mushroom corals (Scleractinia, Fungiidae) of Espiritu Santo (Vanuatu, West Pacific), with the description of a new species. Zoosystema 34(2): 429–443. doi: 10.5252/z2012n2a14

[B41] Hoeksema B (2012b) Forever in the dark: the cave-dwelling azooxanthellate reef coral *Leptoseris troglodyta* sp. n. (Scleractinia, Agariciidae). ZooKeys 228: 21–37. doi: 10.3897/zookeys.228.3798 23166468 10.3897/zookeys.228.3798 PMC3487639

[B42] Hoeksema BW (2012c) Distribution patterns of mushroom corals (Scleractinia: Fungiidae) across the Spermonde Shelf, South Sulawesi. Raffles Bulletin of Zoology 60: 183–212.

[B43] Hoeksema BW (2013) In search of the Asian-Pacific centre of maximum marine biodiversity: explanations from the past and present. Galaxea, Journal of Coral Reef Studies 15 (Supplement): 1–8.

[B44] Hoeksema BW (2014) The “*Fungia patella* group” (Scleractinia, Fungiidae) revisited with a description of the mini mushroom coral *Cycloseris boschmai* sp. n. ZooKeys 371: 57–84. doi: 10.3897/zookeys.371.6677 24493954 10.3897/zookeys.371.6677 PMC3909799

[B45] Hoeksema BW (2015) Latitudinal species diversity gradient of mushroom corals off eastern Australia: a baseline from the 1970s. Estuarine, Coastal and Shelf Science. doi: 10.1016/j.ecss.2015.05.015

[B46] Hoeksema BW Lane DJW (2014) The mushroom coral fauna (Scleractinia: Fungiidae) of Brunei Darussalam (South China Sea) and its relation to the Coral Triangle. Raffles Bulletin of Zoology 62: 566–580.

[B47] Hoeksema BW Moka W (1989) Species assemblages and ecomorph variation of mushroom corals (Scleractinia: Fungiidae) related to reef habitats in the Flores Sea. Netherlands Journal of Sea Research 23: 149–160. doi: 10.1016/0077-7579(89)90009-4

[B48] Hoeksema BW Yeemin T (2011) Late detachment conceals serial budding by the free-living coral *Fungia fungites* in the Inner Gulf of Thailand. Coral Reefs 30: 975. doi: 10.1007/s00338-011-0784-9

[B49] Hoeksema BW Dautova TN Savinkin OV Vo ST Hoang XB Phan KH Hoang TD (2010) The westernmost record of the coral *Leptoseris kalayaanensis* in the South China Sea. Zoological Studies 49: 325.

[B50] Huang D Benzoni F Arrigoni R Baird AH Berumen ML Bouwmeester J Chou LM Fukami H Licuanan WY Lovell ER Meier R Todd PA Budd AF (2014) Towards a phylogenetic classification of reef corals: the Indo-Pacific genera *Merulina*, *Goniastrea* and *Scapophyllia* (Scleractinia, Merulinidae). Zoologica Scripta 43: 531–548. doi: 10.1111/zsc.12061

[B51] Huang D Licuanan WY Hoeksema BW Chen CA Ang PO Huang H Lane DJW Vo ST Waheed Z Affendi YA Yeemin T Chou LM (2015) Extraordinary diversity of reef corals in the South China Sea. Marine Biodiversity 45: 157–168. doi: 10.1007/s12526-014-0236-1

[B52] Huelsenbeck JP Ronquist F (2001) MRBAYES: Bayesian inference of phylogeny. Bioinformatics 17: 754–755. doi: 10.1093/bioinformatics/17.8.754 11524383 10.1093/bioinformatics/17.8.754

[B53] Hutchison CS Vijayan VR (2010) What are the Spratly Islands? Journal of Asian Earth Sciences 39: 371–385. doi: 10.1016/j.jseaes.2010.04.013

[B54] Ismail G Shizuri Y Oakley S Pilcher N Miyachi S (1998) Evaluation of marine communities: Their potential as bioresources. In: Proceedings of The Tokyo International Forum on Conservation and Sustainable Use of Tropical Bioresources, Tokyo, Japan, 9-10 November 1998 New Energy and Industrial Technology Development Organization (NEDO) and Japan Bioindustry Association (JBA), Japan, 84–110.

[B55] Kahng SE Maragos JE (2006) The deepest, zooxanthellate scleractinian corals in the world? Coral Reefs 25: 254. doi: 10.1007/s00338-006-0098-5

[B56] Karlson RH Cornell HV (1998) Scale-dependent variation in local vs. regional effects on coral species richness. Ecological Monographs 68: 259–274. doi: 10.1890/0012-9615(1998)068[0259:SDVILV]2.0.CO;2

[B57] Kitahara MV Cairns SD Stolarski J Blair D Miller DJ (2010) A comprehensive phylogenetic analysis of the Scleractinia (Cnidaria, Anthozoa) based on mitochondrial CO1 sequence data. PLoS ONE 5(7): . doi: 10.1371/journal.pone.0011490 10.1371/journal.pone.0011490 PMC2900217 20628613

[B58] Kitahara MV Stolarski J Miller DJ Benzoni F Stake J Cairns SD (2012) The first modern solitary Agariciidae (Anthozoa, Scleractinia) revealed by molecular and microstructural analysis. Invertebrate Systematics 26: 303–315. doi: 10.1071/IS11053

[B59] Kitano YF Benzoni F Arrigoni R Shirayama Y Wallace CC Fukami H (2014) A phylogeny of the family Poritidae (Cnidaria, Scleractinia) based on molecular and morphological analyses. PLoS ONE 9(5): . doi: 10.1371/journal.pone.0098406 10.1371/journal.pone.0098406 PMC4037213 24871224

[B60] Ku Yaacob KK Ibrahim M (2004) Temperature, salinity and density properties of the southeastern South China Sea: Pulau Layang Layang area. In: Abdullah MP (Ed.) Marine Biodiversity of Pulau Layang-Layang Malaysia, Fisheries Research Institute, Department of Fisheries Malaysia, 89–102. http://www.fri.gov.my/marsal/penerbitan/temperature1.pdf

[B61] Lamarck JBP (1816) Histoire naturelle des Animaux sans Vertèbres, présentant les caractères généraux et particuliers de ces animaux, leur distribution, leurs classes, leurs familles, leurs genres, et la citation des principales espèces qui s’y rapportent; précédée d’une Introduction offrant la Détermination des caractères essentiels de l’Animal, sa distinction du Végétal et des autres corps naturels, enfin, l’exposition des principes fondamentaux de la Zoologie. Déterville & Verdière, Paris.

[B62] Lane DJW (2011) Bleaching and predation episodes on Bruneian coral reefs. Scientia Bruneiana 12: 51–58.

[B63] Lane DJW (2012) *Acanthaster planci* impact on coral communities at permanent transect sites on Bruneian reefs, with a regional overview and a critique on outbreak causes. Journal of the Marine Biological Association of the United Kingdom 92: 803–809. doi: 10.1017/S0025315411000890

[B64] Licuanan WY Aliño PM (2009) *Leptoseris kalayaanensis* (Scleractinia: Agariciidae), a new coral species from the Philippines. Raffles Bulletin of Zoology 57: 1–4.

[B65] Lourey MK Ryan DAJ Miller IR (2000) Rates of decline and recovery of coral cover on reefs impacted by, recovering from and unaffected by crown-of-thorns starfish *Acanthaster planci*: a regional perspective on the Great Barrier Reef. Marine Ecology Progress Series 196: 179–186. doi: 10.3354/meps196179

[B66] Luck DG Forsman ZH Toonen RJ Leicht SJ Kahng SE (2013) Polyphyly and hidden species among Hawai‘i’s dominant mesophotic coral genera, *Leptoseris* and *Pavona* (Scleractinia: Agariciidae). PeerJ 1: . doi: 10.7717/peerj.132 10.7717/peerj.132 PMC3747016 24032091

[B67] Maragos J Jokiel P (1986) Reef corals of Johnston Atoll: one of the world’s most isolated reefs. Coral Reefs 4: 141–150. doi: 10.1007/BF00427935

[B68] Mohamed MIH Rahman RA Abdullah MP (1994) Impact of development on Pulau Layang-Layang coral reefs. In: Sudara S Wilkinson CR Chou LM (Eds) Proceedings, Third ASEAN-Australia Symposium on Living Coastal Resources, Volume 2 Research Papers, May 1994 Chulalongkorn University, Bangkok, 35–40.

[B69] Moran PJ (1986) The *Acanthaster* phenomenon. Oceanography and Marine Biology, An Annual Review 24: 379–480.

[B70] Morton B Blackmore G (2001) South China Sea. Marine Pollution Bulletin 42: 1236–1263. doi: 10.1016/S0025-326X(01)00240-5 11827109 10.1016/s0025-326x(01)00240-5

[B71] Musa G Kadir SLSA Lee L (2006) Layang Layang: an empirical study on SCUBA divers’ satisfaction. Tourism in Marine Environments 2(2): 89–102.

[B72] Nasrulhakim M Daud A Mushidi H (2010) Study of population, distribution and proposed method to control Crown-of-thorns (COT) starfish species outbreak in the waters of Layang-Layang Island, Malaysia. Unpublished report, 15 pp.

[B73] Nishihira M Veron JEN (1995) Corals of Japan. Kaiyusha Publishers, Tokyo.

[B74] Ormond RFG Hanscomb NJ Beach DH (1976) Food selection and learning in the crown-of-thorns starfish *Acanthaster planci* (L). Marine Behaviour and Physiology 4: 93–105. doi: 10.1080/10236247609386944

[B75] Osborne K Dolman AM Burgess SC Johns KA (2011) Disturbance and the dynamics of coral cover on the Great Barrier Reef (1995–2009). PLoS ONE 6(3): doi: 10.1371/journal.pone.0017516 10.1371/journal.pone.0017516 PMC3053361 21423742

[B76] Penn O Privman E Ashkenazy H Landan G Graur D Pupko T (2010a) GUIDANCE: a web server for assessing alignment confidence scores. Nucleic Acids Research 38: W23–W28. doi: 10.1093/nar/gkq443 20497997 10.1093/nar/gkq443 PMC2896199

[B77] Penn O Privman E Landan G Graur D Pupko T (2010b) An alignment confidence score capturing robustness to guide-tree uncertainty. Molecular Biology and Evolution 27: 1759–1767. doi: 10.1093/molbev/msq06 20207713 10.1093/molbev/msq066 PMC2908709

[B78] Pichon M (2007) Scleractinia of New Caledonia: check list of reef dwelling species. In: Payri C Richer De Forges B (Eds) Compendium of marine species of New Caledonia. Documents Scientifiques et Techniques IRD, Nouméa 117(2): 149–157.

[B79] Pichon M Benzoni F (2007) Taxonomic re-appraisal of zooxanthellate scleractinian corals in the Maldive Archipelago. Zootaxa 1441: 21–33. doi: 10.1353/psc.2007.0011

[B80] Pilcher N Cabanban AS (2000) The status of coral reefs in eastern Malaysia. Global Coral Reef Monitoring Network (GCRMN) Report. Australia Institute of Marine Science, Townsville, 63 pp.

[B81] Pilcher N Oakley S Ismail G (1999) Layang Layang a drop in the ocean. Natural History Publications, Kota Kinabalu, 126 pp.

[B82] Pillai CSG Scheer G (1976) Report on the stony corals from the Maldive Archipelago. Zoologica, Stuttgart 126: 1–83, Pl. 1–32.

[B83] Pratchett MS (2007) Feeding preferences of *Acanthaster planci* (Echinodermata: Asteroidea) under controlled conditions of food availability. Pacific Science 61: 113–120.

[B84] Pratchett MS (2010) Changes in coral assemblages during an outbreak of *Acanthaster planci* at Lizard Island, northern Great Barrier Reef (1995-1999). Coral Reefs 29: 717–725. doi: 10.1007/s00338-010-0602-9

[B85] Pratchett MS Shenk TJ Baine M Syms C Baird AH (2009) Selective coral mortality associated with outbreaks of *Acanthaster planci* L. in Bootless Bay, Papua New Guinea. Marine Environmental Research 67: 230–236. doi: 10.1016/j.marenvres.2009.03.001 19327821 10.1016/j.marenvres.2009.03.001

[B86] Ronquist F Huelsenbeck JP (2003) MRBAYES 3: Bayesian phylogenetic inference under mixed models. Bioinformatics 19: 1572–1574. doi: 10.1093/bioinformatics/btg180 12912839 10.1093/bioinformatics/btg180

[B87] Ronquist F Teslenko M van der Mark P Ayres DL Darling A Höhna S Larget B Liu L Suchard MA Huelsenbeck JP (2012) MrBayes 3.2: efficient Bayesian phylogenetic inference and model choice across a large model space. Systematic Biology 61: 539–542. doi: 10.1093/sysbio/sys029 22357727 10.1093/sysbio/sys029 PMC3329765

[B88] Rooney J Donham E Montgomery A Spalding H Parrish F Boland R Fenner D Gove J Vetter O (2010) Mesophotic coral ecosystems in the Hawaiian Archipelago. Coral Reefs 29: 36–367. doi: 10.1007/s00338-010-0596-3

[B89] Saadon N Lim PK Snidvong A Rojana-Anawat P (1999) Physical characteristics of watermass in the South China Sea, area II: Sarawak, Sabah and Brunei Darussalam waters. In: Proceedings of the second technical seminar on marine fishery resources survey in the South China Sea Area II: Sarawak, Sabah and Brunei Darussalam waters, Kuala Lumpur, Malaysia, 14-15 December 1998 Southeast Asian Fisheries Development Center, Samutprakan, Thailand, 1–22.

[B90] Sahari A Ilias Z Sulong N Ibrahim K (2004) Giant clam species and distribution at Pulau Layang-Layang, Sabah. In: Abdullah MP (Ed.) Marine Biodiversity of Pulau Layang-Layang Malaysia, Fisheries Research Institute, Department of Fisheries Malaysia, 25–28. http://www.fri.gov.my/marsal/penerbitan/giantclam1.pdf

[B91] Saponari L Montano S Seveso D Galli P (2014) The occurrence of an *Acanthaster planci* outbreak in Ari Atoll, Maldives. Marine Biodiversity. doi: 10.1007/s12526-014-0276-6

[B92] Scheer G Pillai CSG (1983) Report on the stony corals from the Red Sea. Zoologica, Stuttgart 133: 1–198, pls. 1–41.

[B93] Schmitt EF Sluka RD Sullivan-Sealey KM (2002) Evaluating the use of roving diver and transect surveys to assess the coral reef fish assemblage of southeastern Hispaniola. Coral Reefs 21: 216–223. doi: 10.1007/s00338-002-0216-y

[B94] Scott CM Mehrotra R Urgell P (2015) Spawning observation of *Acanthaster planci* in the Gulf of Thailand. Marine Biodiversity (in press). doi: 10.1007/s12526-014-0300-x

[B95] Somerfield PJ Jaap WC Clarke KR Callahan M Hackett K Porter J Lybolt M Tsokos C Yanev G (2008) Changes in coral reef communities among the Florida Keys, 1996–2003. Coral Reefs 27: 951–965. doi: 10.1007/s00338-008-0390-7

[B96] Sukumaran J Holder MT (2010) DendroPy: A Python library for phylogenetic computing. Bioinformatics 26: 1569–1571. doi: 10.1093/bioinformatics/btq228 20421198 10.1093/bioinformatics/btq228

[B97] Svrcula K (2008) Layang Layang diving Malaysia’s last frontier. Marshall Cavendish Editions, Malaysia, 168 pp.

[B98] Swofford DL (2002) PAUP*. Phylogenetic Analysis Using Parsimony (*and Other Methods), Version 4.0. Sinauer Associates, Sunderland, Massachusetts.

[B99] Takabayashi M Carter D Loh W Hoegh_Guldberg O (1998) A coral-specific primer for PCR amplification of the internal transcribed spacer region in ribosomal DNA. Molecular Ecology 7: 928–930.

[B100] Tamura K Stecher G Peterson D Filipski A Kumar S (2013) MEGA6: Molecular Evolutionary Genetics Analysis version 6.0. Molecular Biology and Evolution 30: 2725–2729. doi: 10.1093/molbev/mst197 24132122 10.1093/molbev/mst197 PMC3840312

[B101] Terraneo TI Berumen ML Arrigoni R Waheed Z Bouwmeester J Caragnano A Stefani F Benzoni F (2014) *Pachyseris inattesa* sp. n. (Cnidaria, Anthozoa, Scleractinia): a new reef coral species from the Red Sea and its phylogenetic relationships. ZooKeys 433: 1–30. doi: 10.3897/zookeys.433.8036 25152672 10.3897/zookeys.433.8036 PMC4141178

[B102] Turak E DeVantier L (2011) Field guide to the reef-building corals of Brunei Darussalam. Fisheries Department, Ministry of Industry and Primary Resources, Government of Brunei Darussalam, Brunei Darussalam, 256 pp.

[B103] Veron JEN (2000) Corals of the world. Australian Institute of Marine Science, Townsville.

[B104] Veron JEN Pichon M (1980) Scleractinia of Eastern Australia III. Families Agariciidae, Siderastreidae, Fungiidae, Oculinidae, Merulinidae, Mussidae, Pectiniidae, Caryophylliidae, Dendrophylliidae. Australian Institute of Marine Science Monograph Series 4: 1–422. doi: 10.5962/bhl.title.60646

[B105] Veron JEN DeVantier LM Turak E Green AL Kininmonth S Stafford-Smith MG Peterson N (2009) Delineating the Coral Triangle. Galaxea 11: 91–100. doi: 10.3755/galaxea.11.91

[B106] Veron JEN DeVantier LM Turak E Green AL Kininmonth S Stafford-Smith MG Peterson N (2011) The Coral Triangle. In: Dubinsky Z Stambler N (Eds) Coral reefs: an ecosystem in transition. Springer, Dordrecht, 47–55. doi: 10.1007/978-94-007-0114-4_5

[B107] Waheed Z Hoeksema BW (2013) A tale of two winds: species richness patterns of reef corals around the Semporna peninsula, Malaysia. Marine Biodiversity 43: 37–51. doi: 10.1007/s12526-012-0130-7

[B108] Waheed Z Hoeksema BW (2014) Diversity patterns of scleractinian corals at Kota Kinabalu, Malaysia, in relation to exposure and depth. Raffles Bulletin of Zoology 62: 66–82.

[B109] Waheed Z Ahad BG Jumin R Hussein MAS Hoeksema BW (subm) Corals at the northernmost tip of Borneo: An assessment of species richness patterns and reef benthic assemblages. 10.1371/journal.pone.0146006 PMC4697805 26719987

[B110] Wells JW (1954) Recent corals of the Marshall Islands. United States Geological Survey, Professional Paper 260-I: 385–486.

[B111] White TJ Bruns T Lee S Taylor J (1990) Amplification and direct sequencing of fungal ribosomal RNA genes for phylogenetics. In: Innis MA Gelfand DH Sninsky JJ White TJ (Eds) PCR protocols. A guide to methods and application. Academic Press Inc., San Diego, 315–322. doi: 10.1016/b978-0-12-372180-8.50042-1

[B112] Wood EM Tan BS (1987) Hard Coral. In: The corals reefs of the Bodgaya Islands (Sabah: Malaysia) and Pulau Sipadan. Malayan Nature Journal 40: 189–224.

[B113] Wyrtki K (1961) Physical Oceanography of the Southeast Asian Waters. NAGA Report Volume 2: Scientific Results of Marine Investigations of the South China Sea and the Gulf of Thailand, 1959–1961. University of California, La Jolla, USA, 195 pp.

[B114] Zainuddin I Pauzi MA Abdul Razak L Yazid MY (2000) Preliminary study on the diversity and distribution of sponges at Pulau Layang-layang, Sabah, Malaysia. Malayan Nature Journal 54: 77–86.

[B115] Zakariah ZM Ahmad AR Tan KH Basiron MN Yusoff NA (2007) National report on coral reefs in the coastal waters of the South China Sea – Malaysia. In: National reports on coral reefs in the coastal waters of the South China Sea. UNEP/GEF/SCS Technical Publication 11: 37–54. http://www.unepscs.org/components/com_remository_files/downloads/National-Report-Coral-Reefs-Malaysia.pdf

[B116] Zwickl DJ (2006) Genetic algorithm approaches for the phylogenetic analysis of large biological sequence datasets under the maximum likelihood criterion. PhD dissertation, The University of Texas, Austin.

