# Supplementary material for: Scleractinian corals (Fungiidae, Agariciidae and Euphylliidae) of Pulau Layang-Layang, Spratly Islands, with a note on Pavona maldivensis (Gardiner, 1905)
Source: Zookeys. 2015 Aug 12;(517):1–37. doi: 10.3897/zookeys.517.9308 (PMC4547123; doi:10.3897/zookeys.517.9308)
Supplement: Supplementary material 4 — Figure S1 [file zookeys-517-001-s004.pdf]

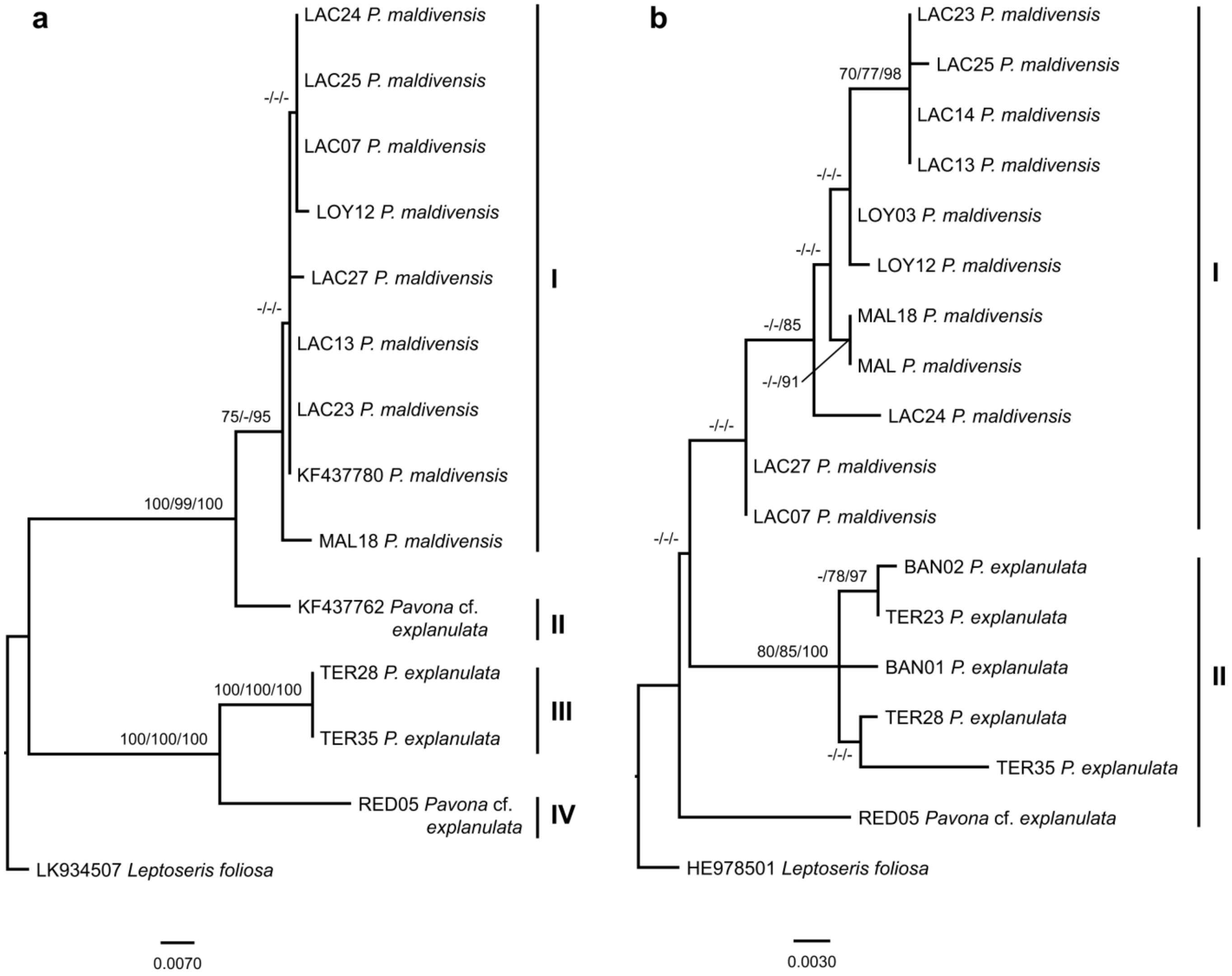

Supplementary file 4: Figure S1. Maximum likelihood phylogram of *Pavona maldivensis* and *P. explanulata*. **a** mitochondrial intergenic spacer between CO1 and 16S-rRna. The sequence alignment consisted of 680 characters with 593 constant, 23 variable and 64 parsimony informative characters, **b** nuclear marker ITS1-5.8S-ITS2, with 680 characters of 649 constant, 14 variable and 17 parsimony informative characters. Support values for maximum likelihood, maximum parsimony (>70) and bayesian posterior probabilities (>80) are given at the nodes. Dashes (-) indicate nodes without statistical support.
