# Supplementary material for: Scleractinian corals (Fungiidae, Agariciidae and Euphylliidae) of Pulau Layang-Layang, Spratly Islands, with a note on Pavona maldivensis (Gardiner, 1905)
Source: Zookeys. 2015 Aug 12;(517):1–37. doi: 10.3897/zookeys.517.9308 (PMC4547123; doi:10.3897/zookeys.517.9308)
Supplement: Supplementary material 5 — Figure S2 [file zookeys-517-001-s005.pdf]

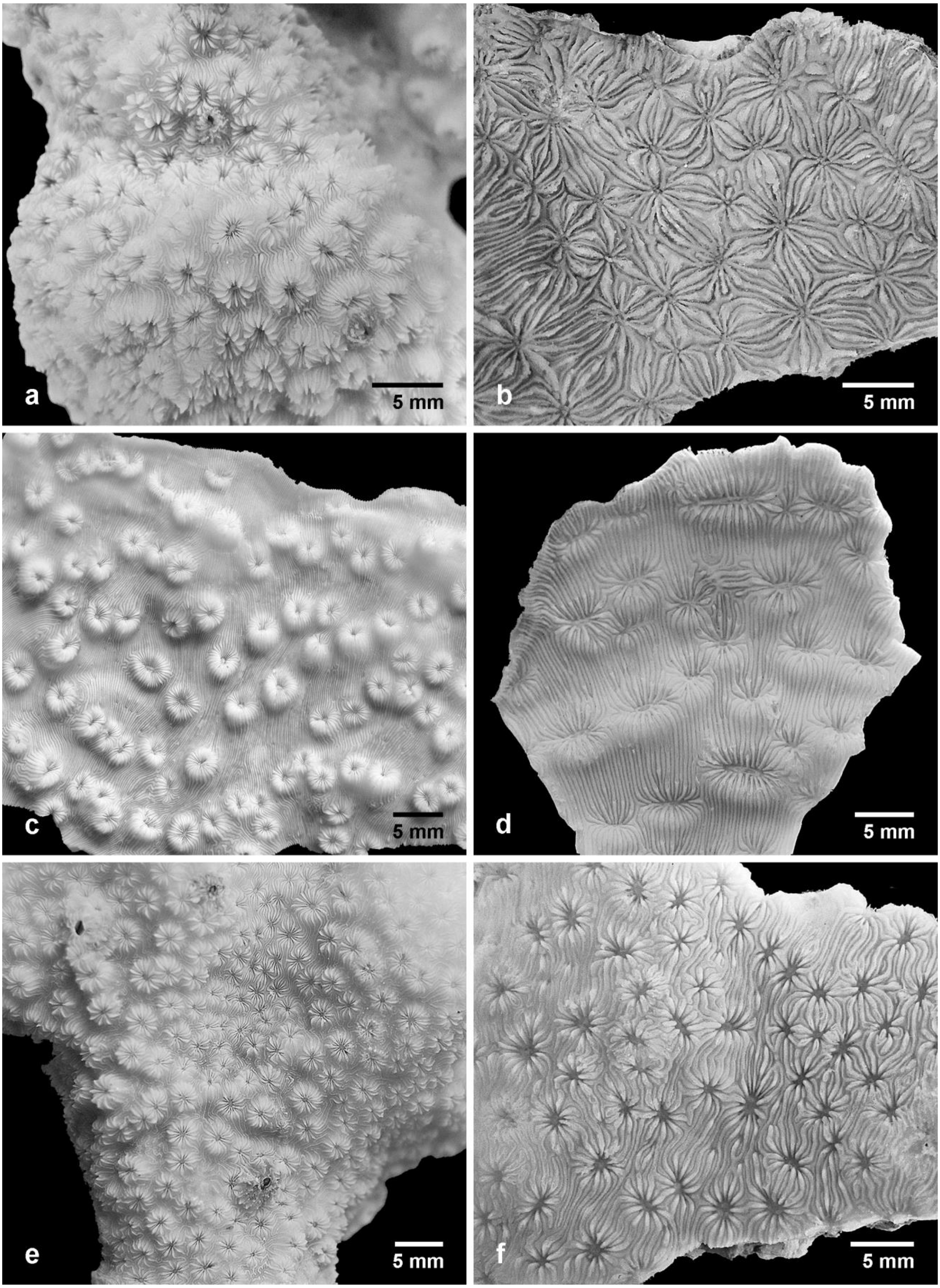

Supplementary file 5: Figure S2. Variation of corallite form and structure in *Pavona maldivensis* (**a**, **c**, **e**), and *P. explanulata* (**b**, **d**, **f**). **a** specimen LAC23 from Layang-Layang, **b** specimen BAN01 from Banggi, North Borneo, **c** specimen LOY12 from Loyalty Islands, **d** specimen BAN02 from Banggi, North Borneo, **e** specimen MAL from the Maldives, and **f** specimen TER28 from Ternate, Indonesia.
